# BILBO1 Is a Scaffold Protein of the Flagellar Pocket Collar in the Pathogen *Trypanosoma brucei*

**DOI:** 10.1371/journal.ppat.1004654

**Published:** 2015-03-30

**Authors:** Célia Florimond, Annelise Sahin, Keni Vidilaseris, Gang Dong, Nicolas Landrein, Denis Dacheux, Anna Albisetti, Edward H. Byard, Mélanie Bonhivers, Derrick R. Robinson

**Affiliations:** 1 University Bordeaux, Microbiologie Fondamentale et Pathogenicité, Bordeaux, France; 2 CNRS, Microbiologie Fondamentale et Pathogénicité, UMR 5234, Bordeaux, France; 3 Max F. Perutz Laboratories, Medical University of Vienna, Vienna, Austria; 4 Institut Polytechnique de Bordeaux, Microbiologie Fondamentale et Pathogénicité, UMR-CNRS 5234, Bordeaux, France; University of California, Los Angeles, UNITED STATES

## Abstract

The flagellar pocket (FP) of the pathogen *Trypanosoma brucei* is an important single copy structure that is formed by the invagination of the pellicular membrane. It is the unique site of endo- and exocytosis and is required for parasite pathogenicity. The FP consists of distinct structural sub-domains with the least explored being the annulus/horseshoe shaped flagellar pocket collar (FPC). To date the only known component of the FPC is the protein BILBO1, a cytoskeleton protein that has a N-terminus that contains an ubiquitin-like fold, two EF-hand domains, plus a large C-terminal coiled-coil domain. BILBO1 has been shown to bind calcium, but in this work we demonstrate that mutating either or both calcium-binding domains prevents calcium binding. The expression of deletion or mutated forms of BILBO1 in trypanosomes and mammalian cells demonstrate that the coiled-coil domain is necessary and sufficient for the formation of BILBO1 polymers. This is supported by Yeast two-hybrid analysis. Expression of full-length BILBO1 in mammalian cells induces the formation of linear polymers with comma and globular shaped termini, whereas mutation of the canonical calcium-binding domain resulted in the formation of helical polymers and mutation in both EF-hand domains prevented the formation of linear polymers. We also demonstrate that in *T. brucei* the coiled-coil domain is able to target BILBO1 to the FPC and to form polymers whilst the EF-hand domains influence polymers shape. This data indicates that BILBO1 has intrinsic polymer forming properties and that binding calcium can modulate the form of these polymers. We discuss whether these properties can influence the formation of the FPC.

## Author Summary

*Trypanosoma brucei* avoids destruction by, in part, changing its surface glycoprotein coat, which is trafficked onto the cell surface *via* an invagination of the cell surface called the flagellar pocket. The pocket is essential for pathogenicity. The distal membrane of the pocket is anchored to a cytoskeleton structure called the flagellar pocket collar (FPC). The FPC is a ring/horseshoe shaped structure, which itself is attached to the single copy flagellum of the parasite. How the “ring” shape of the collar is formed is not understood. Moreover, the only known protein component of the FPC is the protein BILBO1. BILBO1 is modular and has a distinct N-terminal domain, two EF-hand calcium-binding domains and a large C-terminal coiled-coil domain. Here we demonstrate that mutating the EF hand domains prevent calcium binding and that the coiled-coil domain is not only sufficient to target to the collar, but can also form polymers in mammalian cells. Mutating either or both calcium-binding domains of BILBO1 influences polymer formation and type when expressed in mammalian and trypanosome cells. Our premise is that BILBO1 has intrinsic polymer forming properties that are essential for the flagellar pocket collar making the pocket a target for intervention.

## Introduction

*Trypanosoma brucei* is an important parasitic protozoan that is the etiological agent of sleeping sickness in sub-Saharan Africa. Related parasites are responsible for Chagas disease and Leishmaniasis in South America and many tropical countries [1,2,3]. At the G1 stage of the *T*. *brucei* cell cycle a single flagellum exits the cell through the flagellar pocket (FP), a structure that is located in the posterior end of the cell. The FP functions as the exclusive site for endo- and exocytosis, and has been shown to be an essential component of membrane trafficking and recycling [4,5,6]. In these roles the FP is essential for parasite virulence, because *T*. *brucei* must survive within both the gut and salivary glands of the tsetse fly as well as in the bloodstream of the mammalian host. Thus the FP is also most likely a functional design to sequester important parasite surface receptors away from detection by the host’s innate immune system [5,7].

The tight coupling between the FP, the flagellum, and the cytoplasmic membranes has been well established in recent studies where work on the *T*. *brucei* FP and associated cytoskeleton suggest that new FP biogenesis is precisely timed to coordinate with flagellum duplication and segregation [6,8]. Electron microscopic imaging and tomography clearly illustrate that a cytoskeletal structure called the flagellar pocket collar (FPC), a horse-shoe/annular structure, of approximately 500–800 nm in diameter, in *T*. *brucei*, is present at the exit point of the flagellum [4,6,9]. The FPC surrounds the flagellum and is also attached to the sub-pellicular microtubule cytoskeleton [6,8], but it is not known how it is attached, nor is it apparent how the FPC always forms its characteristic shape around a newly formed flagellum. Recently, an important structure called the bilobe has been identified as being closely associated with the FPC. The bilobe is considered to be a Golgi-linked structure that contains Centrin 2, and numerous other proteins [9,10,11]. The intimate relationship between the FP-flagellum, the bilobe and Golgi [12] suggests that at least some of these structures are physically linked [11].

As we demonstrated previously, BILBO1 is syntenic and essential for biogenesis of the FPC [8]. RNAi knockdown of BILBO1 in *T*. *brucei* disrupts the formation of the FPC, inhibits the biogenesis of important cytoskeleton structures, induces severe perturbation of the endo-membrane system, cell cycle arrest, and is ultimately lethal. BILBO1 is the first, and to date the only, FPC molecular component identified that is required for FPC and FP biogenesis, which makes it a potentially important target for intervention against kinetoplastids [8].

Recently, the three-dimensional structure of BILBO1 N-terminal domain was solved and revealed that it contains an unexpected ubiquitin-like fold with a conserved surface patch [13,14]. Mutation of the patch was lethal when expressed in *T*. *brucei* procyclic forms suggesting that there are important interactions between the patch and other BILBO1 protein partners [13,14]. Using electron microscopy Vidilaseris *et al.*, demonstrated that the EF-hand domains of BILBO1 change their conformation upon calcium binding, and the coiled-coil domain can form anti-parallel dimers, which can then form linear polymers *via* the C-terminal leucine zipper. Further, they demonstrated that these filaments can condense into fibers through lateral interactions [15].

In this study, we turn to an analysis of BILBO1 protein as an essential candidate of the FPC scaffold. Our overall objective was to identify the molecular role of BILBO1 in FPC formation. The primary and secondary structures of BILBO1 do not predict a specific function, and the protein does not appear to have any obvious membrane-targeting domains, but it does possess two predicted EF-hand calcium-binding domains (aa 185–213 and aa 221–249). It also has a large coiled-coil (CC) domain (aa 263–566), which is involved in protein-protein interactions [15]. Based on our hypothesis that BILBO1 is the FPC scaffold, we decided to determine 1) if BILBO1 can form polymers *in vivo*, 2) what domain(s) of the protein is(are) involved in polymer formation. We approached these questions with the following experiments; 1) Identification of functional domains involved in BILBO1-BILBO1 interaction by yeast-two hybrid analysis 2), test for intrinsic polymer formation properties of BILBO1 using a heterologous mammalian expression system and 3), characterization of these properties in the parasite.

We demonstrate in this work that BILBO1 can form polymers *in vivo* and propose that these may have important implications for the formation of the annulus/horseshoe of the FPC. The results we report here point to a substantial role for BILBO1 in forming the structural scaffold for FPC biogenesis and maintenance.

## Results

### BILBO1-BILBO1 interaction is *via* the coiled-coil domain

**Fig 1:**
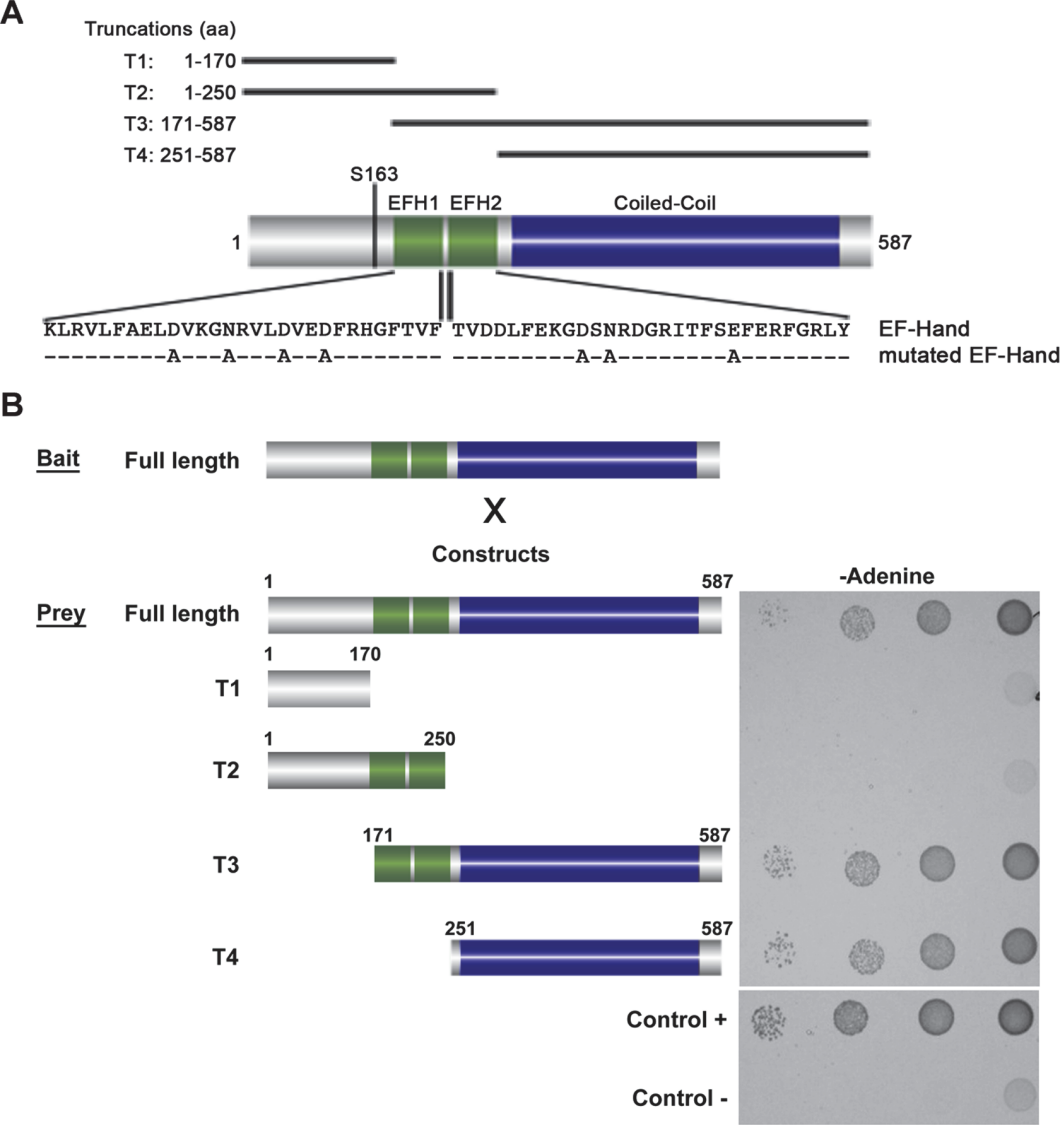
Schematic diagram of BILBO1 primary structure and yeast two-hybrid identification of its interaction domains. (A) BILBO1 has two predicted EF-hand calcium-binding domain EFH1 and EFH2, from amino acids Lysine 185 to Tyrosine 249, and a long domain predicted as coiled-coil. Serine 163 is phosphorylated *in vivo*. The main truncations used in this study are shown schematically and labelled as T1-T4. The mutations in the EF-hand calcium binding domains are indicated below the wild-type amino acid sequence. (B) Bait (BILBO1 full-length) and prey interactions were tested by drop tests on adenine-free selective medium. Full-length BILBO1 interacts with full-length BILBO1, whilst truncations T1 and T2 do not interact with full-length BILBO1. T3 and T4 truncations interactions were positive, demonstrating that the coiled-coil domain is required for BILBO1-BILBO1 interaction.

Yeast-Two-Hybrid (Y2H) analysis has been used to test interactions between soluble proteins, but also between polymer forming proteins [16,17,18]. We used this technique to test if BILBO1 could form homo-polymers and, if yes, identify the domains that are involved in this interaction. For this study several BILBO1 truncations were constructed and were named as follows; T1 for the N-terminal domain (aa 1–170), T2 for the N-terminal domain including both EF-hand calcium-binding domains (aa 1–250), T3 for the CC domain including the two EF-hand domains up to the C-terminus (aa 171–587), and T4 for the CC domain up to the C-terminus (aa 251–587) (Fig. 1A). BILBO1 and its truncations tested negative for toxicity and auto-activation in these Y2H experiments.

Interactions were visualised using two auxotrophic assays with similar results (minus Adenine is shown in Fig. 1B). As yeast growth was observed when full-length BILBO1 construct was tested (Fig. 1B), our assays show that there is a BILBO1 x BILBO1 interaction (full-length x full-length). Further, they show that the coiled-coil domain is required for the interaction (full-length x T3, or full-length x T4), and that neither the N-terminal domain (T1 and T2) nor the EF-hand domains 1 and 2 are required in this interaction *per se* (Fig. 1B).

### Both BILBO1 EF-hand domains bind calcium

BILBO1 has two putative calcium-binding EF-hand domains that are located between the N-terminus and the coiled-coil domain. *In silico* analysis of these EF-hand domains indicate that domain1 is non-canonical (12-residue loop—aa 194–205, but contains a lysine at position +Y, whereas this is typically aspartic acid or asparagine), but domain 2 is canonical (12-residue loop—aa 230–241) [19,20,21]. The 3D structure of the N-terminus of BILBO1 has been solved, but the domain analyzed does not contain the EF-hands [14].

To characterize further the EF-hand domains, we tested the wild-type and mutated forms for calcium binding properties using isothermal calorimetry (ITC) [22]. The DNA sequence encoding the EF-hand domains (amino acid residues 177–250) of wild-type BILBO1 were cloned to incorporate a N-terminal maltose-binding tag plus a 10 × histidine tag (MBP-His_10_). This construct was used as a template to make the mutated forms of the EF-hand domains. For the mutant forms the following amino acid substitutions were created: Mutated EF-hand 1 (mEFH1: D194A, N198A, D202A, and D205A), and mutated EF-hand 2 (mEFH2: D230A, N232A, and E241A), or both mutated EF-hands (mEFH1+2). The amino acids selected for mutation were based on published analysis of EF-hand function by Gifford *et al.*,[19].

**Fig 2:**
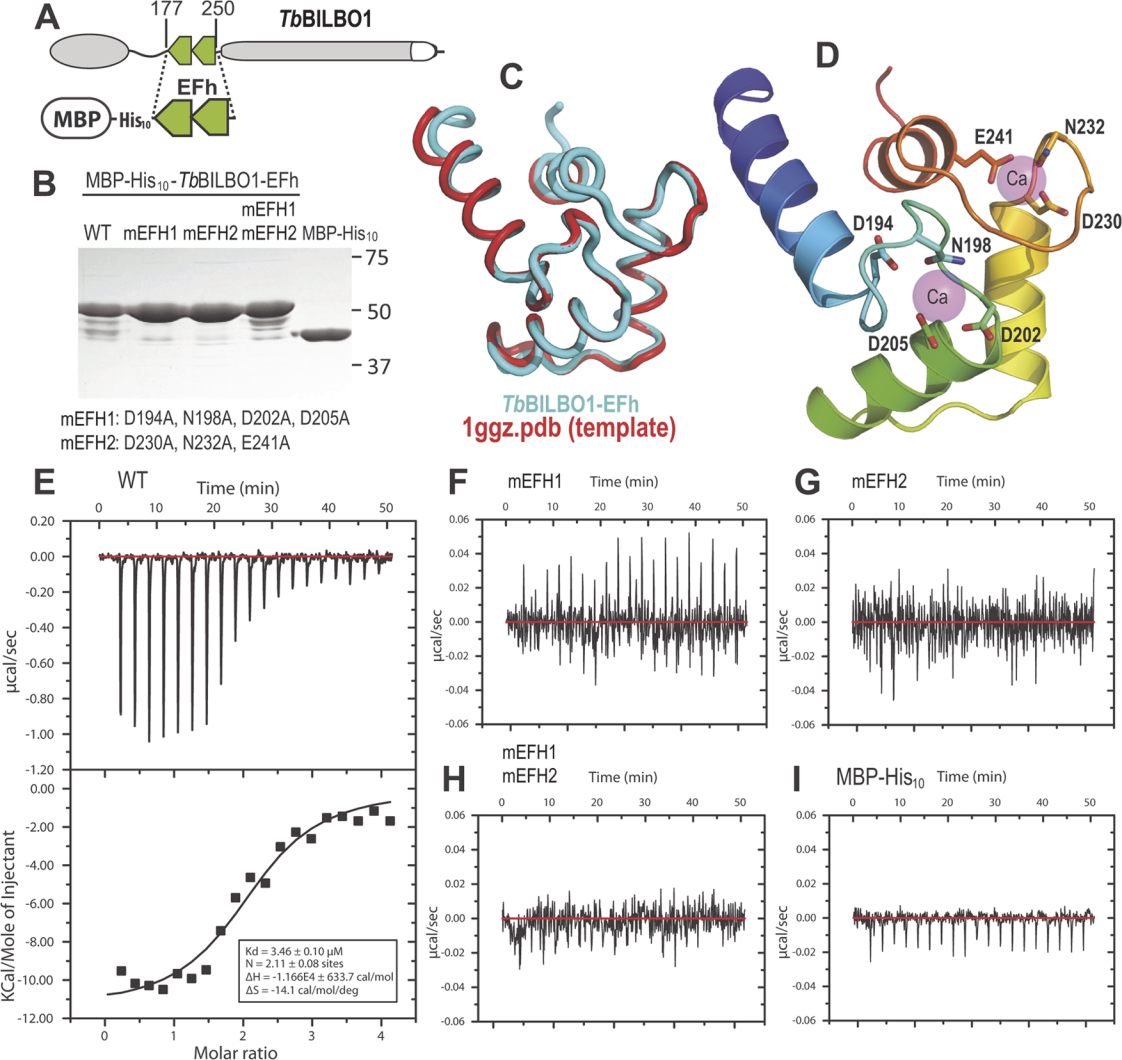
The conserved residues in the loops of the EF-hand domains are required for calcium binding. (A) Schematic depicting the *Tb*BILBO1-EFh (residues 177–250) expression construct used in the *in vitro* calcium-binding experiments. The protein was expressed with an N-terminal tag containing maltose-binding protein (MBP) together with a 10 × histine linker. (B) Purified proteins of wild-type (WT) and three mutants of MBP-His_10_-TbBILBO1-EFh were analyzed by SDS-PAGE and stained using Coomassie blue. The minor bands are degradation products. The last lane shows purified MBP-His_10_ alone. (C) Superimposition of the *Tb*BILBO1-EFh model onto the modeling template 1ggz.pdb, (a human epithelial cell calmodulin-like protein). The two proteins share 30% identities and 47% similarities in their primary sequences. (D) Ribbon diagram of the *Tb*BILBO1-EFh derived from homology-based modeling shown in (C). The structure is color-ramped from blue at the N-terminus to red at the C-terminus. Conserved D/E/N residues in the loops, which are predicted to coordinate calcium binding, are shown as sticks. The two calcium sites from the modeling template (1ggz.pdb) are shown as semi-transparent magenta spheres. (E) ITC titration and fit curve for the wild-type *Tb*BILBO1-EF-hand domains. N, which represents the molar ratio between Ca^2+^ and the protein, was determined to be approximately 2, suggesting that both EF-hand domains of *Tb*BILBO1 bind calcium. (F-H) ITC titration results for mEFH1, mEFH2, and mEFH1+2. None of the three mutants were able to bind calcium. (I) ITC titration result for the MBP-His_10_ tag. The fusion tag by itself did not bind calcium.

A schematic of BILBO1 is shown in Fig. 2A, whilst Fig. 2B shows the purified proteins on an SDS-PAGE with Coomassie blue staining. The minor bands present under the 50kDa main bands are degradation products. Fig. 2C illustrates a superimposition of BILBO1-EF-hand domains onto the modeling template of the human calmodulin-like protein hCLP (1ggz.pdb) [23]. The two proteins share 30% identity and 47% similarity in their primary sequences, which gave rise a very similar conformation with a root-mean-square deviation (r.m.s.d) of 0.82 Å over 70 aligned residues of the two structures. A ribbon diagram of the BILBO1-EF-hand domains derived from homology-based modeling, together with the two bound calcium ions from the template structure (1ggz.pdb), is shown in Fig. 2D. ITC demonstrated that the wild-type BILBO1 EF-hand domains do bind 2 calcium ions (Fig. 2E, N = 2.11, Kd = 3.46 μM), whereas mutation of either or both EF-hand domains caused loss of calcium binding (Fig. 2F-H). As a negative control, no calcium binding was observed for the MBP fusion tag alone (Fig. 2 I).

### BILBO1 has polymers forming properties *in vivo*

**Fig 3:**
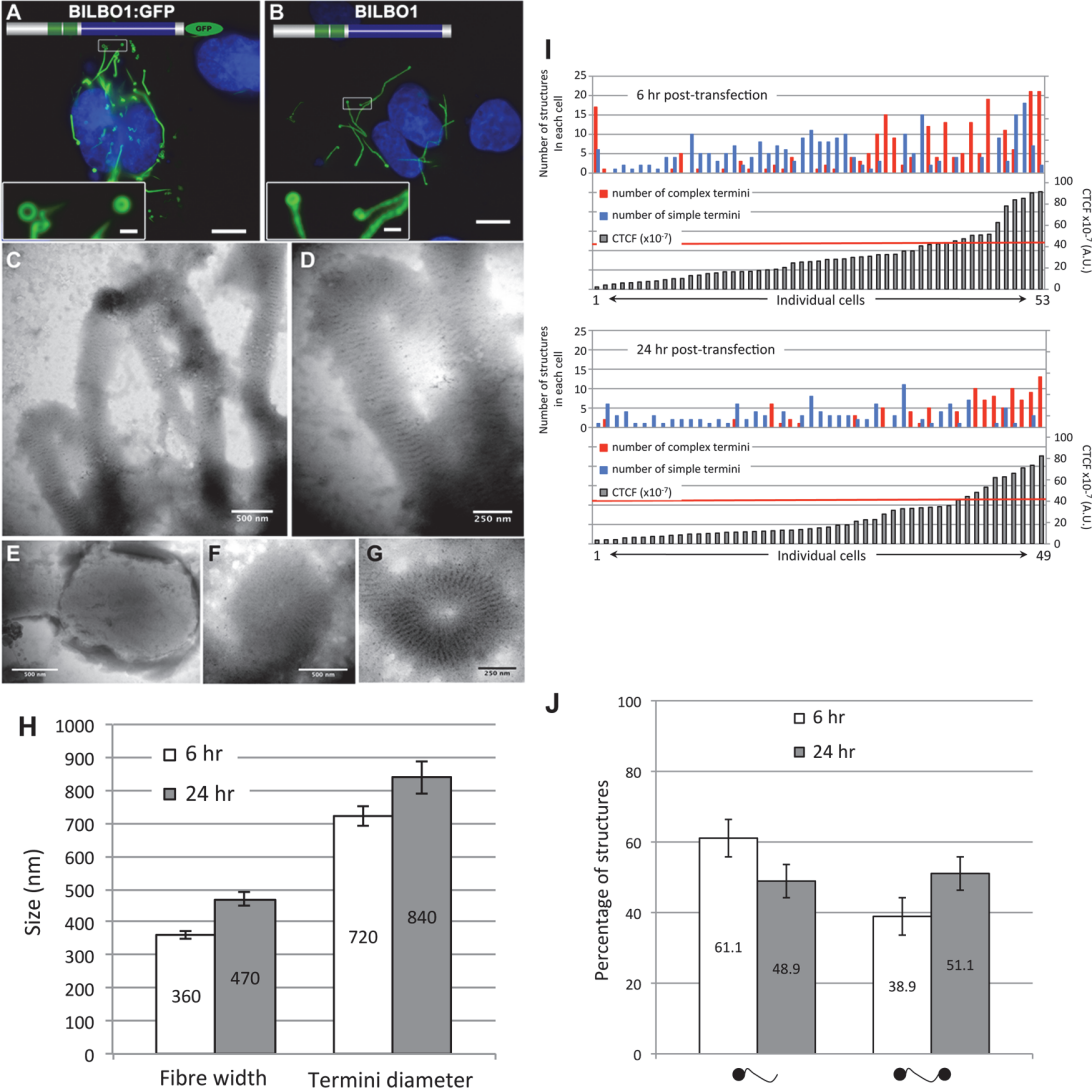
BILBO1 forms polymers in a heterologous system and the coiled-coil domain is required for polymerization. Heterologous expression of BILBO1:GFP (A), or un-tagged BILBO1 (B) in mammalian U-2 OS cells demonstrate that BILBO1 has self-polymerizing properties. After 6 hours post-transfection of BILBO1:GFP the polymers formed were observed by direct GFP fluorescence. After 6 hours post-transfection of un-tagged BILBO1, extracted cells were immuno-labelled with anti-BILBO1 monoclonal antibody. (C-F) Electron micrograph of extracted and negative stained U-2 OS cells after six hours of BILBO1 expression. (G) Electron micrograph of extracted and negative stained U-2 OS cells after six hours of BILBO1:GFP expression. (H) Measurements of fibre width and diameter of termini in U-2 OS cells expressing BILBO1 after six or 24 hours post-transfection and immunolabelled with anti-NTD. (I) Measurements of total fluorescence emitted per cell, as arbitrary units, after a fixed time of acquisition on anti-NTD immunolabelled BILBO1 expressing U-2 OS cells, and number of complex or simple termini in each corresponding cell. The red line indicates the 50% of maximum fluorescence intensity limit. (J) Determination of the percentage of complex polymers with termini at one end (left) or at both ends (right) in BILBO1 U-2 OS expressing cells after six or 24 hours transfection. Scale bars represent 10 μm in A and B, 1 μm in insets, 500 nm in C, E, F, and 250nm in D, G.

To explore the possibility that BILBO1 can form polymers, it was expressed in an *in vivo* heterologous system in the absence of any other parasite-specific proteins. Since BILBO1 has no known mammalian orthologues, we can investigate the polymers formed in U-2 OS cells in detail albeit out of context of the FPC. By transient transfection of U-2 OS cells, we expressed full-length, untagged BILBO1 protein or BILBO1:GFP and analyzed the polymers formed. We acknowledge that there may be other proteins interacting with BILBO1 when expressed in these cells and these can contribute to polymer formation. Nevertheless, direct GFP fluorescence, immuno-labelling of untagged BILBO1 with the anti-BILBO1 monoclonal antibody 5F2B3 or electron microscopy illustrated that expression from six to 24 hours resulted in the formation of long fibrous polymers (Fig. 3A—D also refer to S1 Fig.). Thus, confirming our hypothesis that BILBO1 can indeed form polymers *in vivo*. We noticed the formation of numerous isolated annular structures when BILBO1:GFP was expressed in U-2 OS cells, but since this appears to be a GFP-tag induced artefact, these structures were not analyzed further (Fig. 3A and 3G, and S2 Fig.).

Polymers that started or terminated with globular, or annular/comma, shaped structures, were observed when untagged BILBO1 was expressed (Fig. 3B-F). When viewed by transmission electron microscopy these polymers had transversal striations that were observed after negative staining to have a mean periodicity of 46.9 nm (*n* = 1067, SE± 0.4nm) indicating the formation of highly ordered polymers, (Fig. 3C-F). Interestingly, this is similar to the inter N-termini distance of anti-parallel BILBO1 proteins (40–45nm) observed by Vidilaseris *et al.*, 2014 [15], suggesting a similar assembly arrangement of polymers. A similar periodicity was also observed with the BILBO:GFP construct, but since GFP induces artefacts these striations were not analyzed in detail (Fig. 3G). We have categorized the linear polymers observed by immunofluorescence as “simple”’ or “complex”. Simple polymers have no distinguishable features at their ends, whereas complex polymers have comma or globular structures at one or both ends. To facilitate nomenclature, these structures, from here onwards, will be referred to collectively as “termini”. Fig. 3C-F illustrates termini when visualised by electron microscopy and show that the shapes of these termini are varied. Termini that looked like “spheres” using immunofluorescence are actually dense globular structures when viewed by electron microscopy (Fig. 3E). Formation of these globules maybe due to non-specific aggregation but it is not apparent how comma/annuli are formed, but they appear to be shaped when termini curl back upon themselves.

Polymers could be observed by immunofluorescence microscopy whereas the cell extraction procedure required for electron microscopy removed most of the polymers making them difficult to observe, despite numerous attempts to do so. We therefore measured the width of the BILBO1 fibres and the diameter of the termini using immunofluorescence microscopy. Globules and commas were sometimes difficult to distinguish by immunofluorescence and were therefore grouped together and counted as termini. We also counted the number and type of polymers with complex termini per cell after six and 24 hours of untagged BILBO1 post transfection, (please refer to Table 1 and Fig 3H and 3J).

**Table 1:** Mean termini diameter, fibre width, and termini formation of untagged BILBO1 after expression in U-2 OS cells.

Post transfection time	Fibre width	Termini diameter	Polymers with complex termini / cell
6h	**360 nm** (SE± 10) *n* = 100	**720 nm** (SE± 30) *n* = 110	**61.1%** termini at one end (SE± 5.3) *n* = 23 **38.9%** Termini at both ends (SE± 5.3) *n* = 23
			12h	**470 nm** (SE± 20) *n* = 94	**840 nm** (SE± 50) *n* = 109	-
24h	-	-	**48.9%** termini at one end (SE± 4.7) *n* = 61 **51.1%** Termini at both ends (SE± 4.7) *n* = 61

- indicates not done.

Measurements of BILBO1 fibre width and termini diameter was done using immunofluorescence microscopy. Measurements were made after six and 12 hours, whereas complex termini were measured after six and 24 hours. Fibre width and the number of fibres with termini at both ends increased over time.

From this data and the electron microscopy results we conclude that BILBO1 has the intrinsic capacity to polymerize into organized high order polymers in mammalian cells (in the absence of any other parasite-specific proteins) and these polymers have a tendency to form complex termini at their extremities. Reiterating the results from Fig. 3I this data suggests that the polymerization state of BILBO1 is likely to be highly dependent on the expression level of the protein or local protein concentration.

In addition to these measurements we measured the total fluorescence emitted per cell, as arbitrary units, after a fixed time of acquisition. We used the fluorescence intensity produced by each cell as a marker for protein concentration per cell. We then divided the population into two groups—cells producing less than 50% of the maximum fluorescence recorded, and cells producing more than 50% of the maximum fluorescence recorded.

After six hours of expression of untagged BILBO1 81% of cells produced 50% or **less** of the maximum fluorescence recorded/cell, and 37.2% of these cells contained polymers with only simple termini, whilst 11.6% contained polymers with complex termini and 51.2% contained both types of polymer. 19% of cells produced **more** than 50% of the maximum fluorescence recorded/cell, and 20% of these cells contained polymers with simple termini, whilst 30% contained complex polymers and 50% contained both types of polymer (Fig. 3I).

After 24 hours of expression of untagged BILBO1, 81.7% of cells produced 50% or **less** of the maximum fluorescence recorded/cell and 70% these cells contained polymers with simple termini, whilst 10% contained polymers with complex termini and 20% contained both types of polymer. 18.3% of cells produced **more** than 50% of the maximum fluorescence recorded/cell and 11.1% these cells contained polymers with only simple termini, whilst 44.4% contained polymers with only complex termini and 44.5% contained both types of polymer (Fig. 3I, 24 hours post-transfection). Taken together this data reiterates the hypothesis that the polymerization state of BILBO is likely to be highly dependent on the expression level of the protein.

To investigate whether the BILBO1 polymers were associated with pre-existing or newly formed cytoskeleton structures, U-2 OS cells expressing BILBO1:GFP were additionally labelled for F-actin (phalloidin), intermediate filaments (anti-vimentin), microtubules (anti-tubulin), endoplasmic reticulum Golgi (anti-giantin) and (anti-calnexin). The results presented in S2 Fig. demonstrate that BILBO1:GFP does not co-localize with any of these structures. We conclude that the polymers formed by BILBO1 are not promoted by the interaction with the ER, Golgi or the cytoskeletal structures tested.

### The coiled-coil domain is required for polymer formation *in vivo*

Phosphorylation or dephosphorylation can induce conformational or interaction changes in proteins. Our LC-MS/MS analysis on procyclic form (PCF) whole cell extracts identified one phosphorylated residue (S163) as already described in *T*. *cruzi* bloodstream forms and *T*. *brucei* procyclic forms BILBO1 [24,25]. We postulated that phosphorylation and/or dephosphorylation of serine 163 could induce a conformational change leading to polymer shape changes. Mutation of BILBO1 serine 163 to non-phosphorylatable alanine, or to phosphomimetic aspartic acid, did not influence the type of polymers formed when expressed in U-2 OS cells suggesting that the phosphorylation of serine 163 is not regulating the conformation of BILBO1 or at least polymer formation.

Since phosphorylation of serine 163 did not influence polymer formation we interrogated BILBO1 in detail to identify the domain(s) that permit polymer formation. Thus, we expressed truncated forms of untagged BILBO1 in U-2 OS cells and localized the truncations using immunofluorescence with the monoclonal antibody specific to the CC domain (anti-BILBO1, 5F2B3) [8], or a polyclonal antibody specific to the N-terminal domain of BILBO1 (amino acids 1–110, anti-NTD) [9].

**Fig 4:**
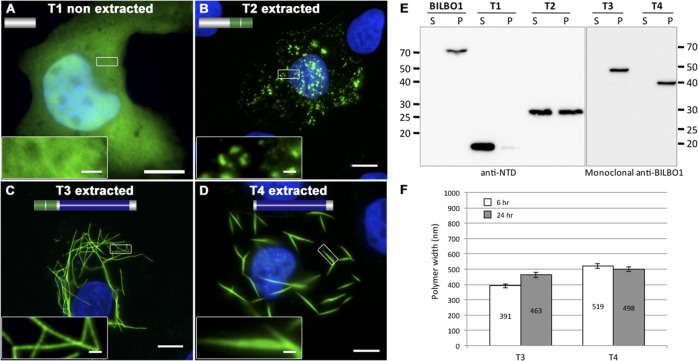
BILBO1 has self-polymerizing properties via its coiled-coil domain. (A-D) Immunofluorescence panels. Heterologous expression of T1 (A), T2 (B), T3 (C), and T4 truncations (D) in U-2 OS cells demonstrate that BILBO1 has self-polymerizing properties *via* its coiled-coil domain. After 6 hours post-transfection, cells expressing the different constructs were immunolabelled using anti-NTD (T1, T2) or 5F2B3 (T3, T4). The inserts in the bottom left of panels represent higher magnifications of structures observed in these panels. Scale bars represent 10 μm, and 1 μm in magnified insets. (E) Western blots probed with anti-NTD (BILBO1, T1, T2) or 5F2B3 (T3, T4) against proteins samples collected from U-2 OS cells after six hours of expression. Samples were collected from pellet and supernatant of detergent extracted cells. BILBO1, T3 and T4 are insoluble. T1 is primarily soluble and T2 is partially soluble. Predicted relative molecular masses (using ExPASy compute pI/Mw) of truncations are T1 = 19kDa, T2 = 25.25kDa, T3 = 48.3kDa, T4 = 39kDa. (F) Measurements of structure width in polymers formed by T3 and T4 truncation at six and 24 hr post-transfection.

The truncation T1 did not produce polymers, and was uniformly distributed throughout the cytoplasm (Fig. 4A). Additionally, T1 labelling could be extracted by mild detergent treatment and is found in the soluble fraction (S) by western-blot (WB) whilst full-length BILBO1 is found in the insoluble fraction (P) (Fig. 4E). T2 was also extracted with detergent treatment, and indicates that T2 is soluble, but can form small, insoluble, punctate aggregates (Fig. 4B, and 4E). The data also suggests that aggregation is likely to be highly dependent on expression level of the protein.

Confirming the Y2H interaction results described earlier, T3 and T4 formed linear polymers (T3) and spindle shaped polymers (T4), but no termini were observed on these fibres (Fig. 4C-D). Similar to full-length BILBO1 these polymers are insoluble and were only found in the pellet fraction by WB (Fig. 4E). Taken together, these results demonstrate that neither the N-terminal domain (corresponding to T1 and T2) nor the EF-hands form linear polymers, but they can influence the type of polymer formed by the CC domain.

We then turned to T3 and T4 and measured the width of induced polymers after six and 24 hours post transfection, (please refer to Table 2 and Fig. 4F). From these measurements we noted an increase in T3 polymer width over time compared to T4, which suggests easier lateral binding of T4 to polymers, and/or may reflect differences in protein expression levels.

**Table 2:** Mean T3 and T4 width after expression in U-2 OS cells.

Post transfection time	T3 mean width	T4 mean width
6h	**391 nm** (SE± 13) *n* = 193	**519 nm** (SE± 16) *n* = 102
24h	**463 nm** (SE± 18) *n* = 155	**498 nm** (SE± 15) *n* = 119

Measurements of the mean T3 and T4 width was done at six and 24 hours of expression in U-2 OS cells. Mean T3 width increased over time whereas T4 did not.

### The EF-hand domains influence BILBO1 polymer formation *in vivo*

**Fig 5:**
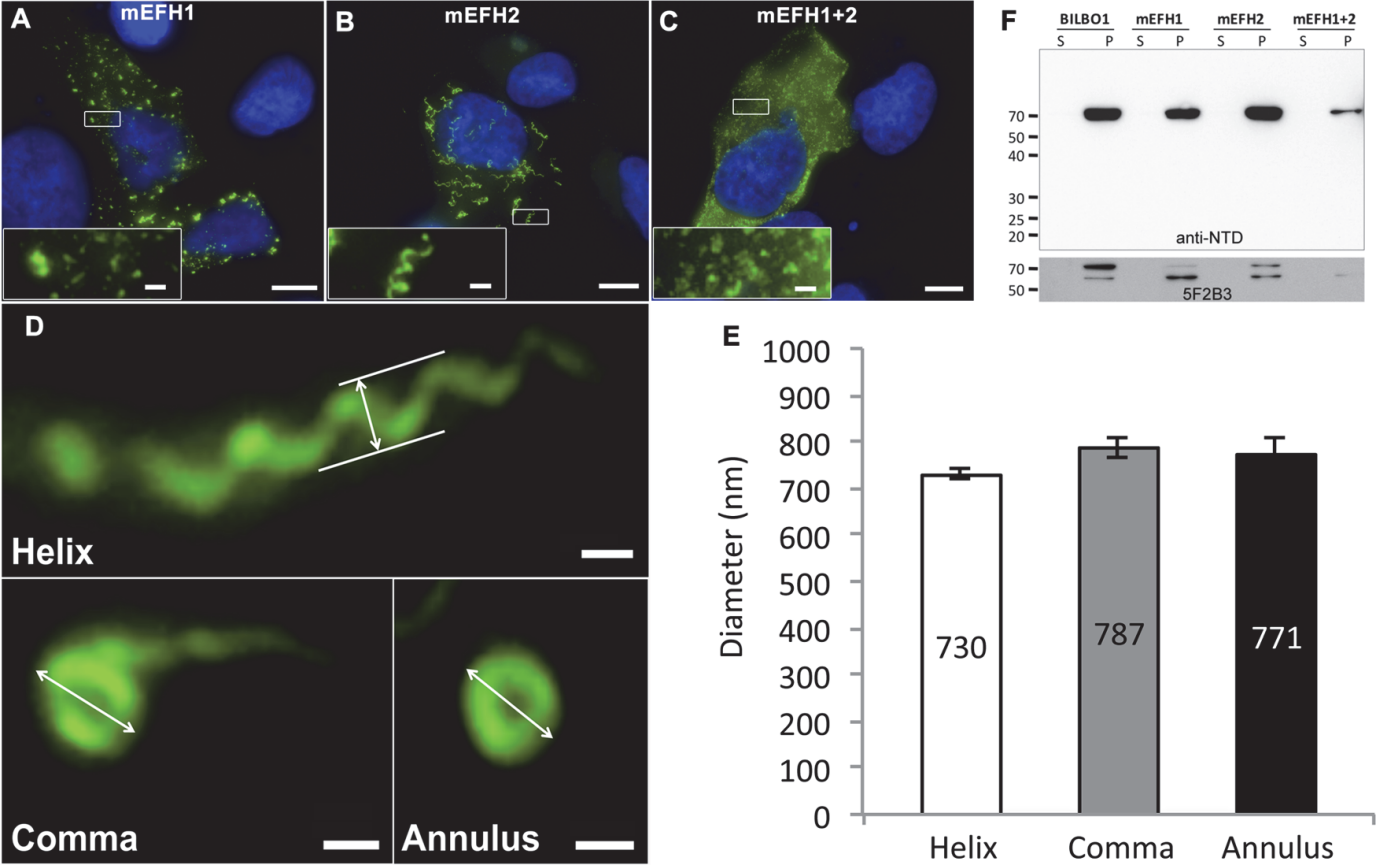
The EF-hand domains modulate the morphology of polymers formed by BILBO1. (A-D) Immunofluorescence panels. EH-Hand motif 1, EF-hand motif 2 and both motifs were mutated by site-directed mutagenesis (see Fig. 1). The resulting mutant proteins mEFH1 (A), mEFH2 (B), and mEFH1+2 (C) were expressed for six hours in U-2 OS cells, and the resulting polymers were immuno-localized using the anti-NTD antibody. The inserts in the bottom left of panels represent higher magnifications of structures observed in these panels. Scale bars represent 10 μm, and scale bars in magnified insets represent 1 μm. (D) Enlarged views of the helix, comma and annulus shaped polymers formed by mEFH2 in U-2 OS cells 24 hours post-transfection. Scale bar represents 500 nm. (E) Helix, comma and annulus shaped polymers have comparable dimensions as shown by the measurements of their respective diameters. (F). A western blot probed with anti-NTD (upper panel) against protein samples collected from U-2 OS cells after six hours of expression of BILBO1, mEFH1, mEFH2 or mEFH1+2 proteins. Samples were collected from pellets and supernatants of detergent extracted cells. BILBO1, mEFH1, mEFH2 and mEFH1+2 were insoluble and subject to degradation as shown with the anti-BILBO1 5F2B3 monoclonal antibody labelling (lower panel) that recognizes the coiled-coil domain.

Since we have established that both BILBO1 EF-hand domains bind calcium and that BILBO1 can form polymers *in vivo*, we wanted to test the potential role of the EF-hand domains in modulating polymers formed in U-2 OS cells. We therefore expressed mutated forms of EF-hand domain 1 (mEFH1), EF-hand domain 2 (mEFH2), or both mutated EF-hand domains (mEFH1+2) in U-2 OS cells (Fig. 5). Mutation of EF-hand domain 1 had a substantial effect on polymerization because the formation of long polymers was abolished, and only small insoluble punctate aggregates were observed (Fig. 5A). Similar aggregates were observed when both EF-hand domains 1 and 2 were mutated (mEFH1+2) (Fig. 5C).

Surprisingly, six-hour expression of mEFH2 resulted in the formation of insoluble helical-like, comma and annular polymers (Fig. 5B, D). Attempts to visualize these by electron microscopy failed due possibly to loss of sample upon cell extraction and/or poor visualization because of the presence of cell debris. Nevertheless, using immunofluorescence we classified them and measured their dimensions six hours post transfection, (please refer to Table 3 and Fig. 5E). The results of these data suggest that EF-hand 2 can radically influence the type of polymer formed, but it also illustrates that these structures have similar diameters even though they can be a helix, comma or annulus in shape (Fig. 5E). Interestingly, the FPC diameter in wild-type *T*. *brucei* cells is 842 nm, which is a diameter comparable to those of the mEFH2-induced structures (please refer to Table 3).

**Table 3:** Mean mEFH2-induced helix, coma and annuli diameter after expression in U-2 OS cells and mean FPC diameter.

Post transfection time	mEFH2			*T*. *brucei* Wild-type FPC diameter
	Helix diameter	Comma diameter	Annuli diameter
6h	**730 nm** (SE± 10) *n* = 95	**787 nm** (SE± 20) *n* = 60	**771 nm** (SE± 40) *n* = 20	**842 nm** (SE± 20) *n* = 71

The mEFH2 helix diameter, comma diameter and annuli diameter were measured after six hours post-transfection. Helix, comma, annuli and FPC diameters all have similar diameters. FPC diameter was measured on wild-type *T*. *brucei* procyclic cells.

Western blots of untagged and mutated BILBO1 proteins, that were expressed in U-2 OS cells, were probed with anti-NTD, and show that all proteins were present in the pellet fraction of extracted cells (∼70 kDa band) and that anti-NTD recognises the full-length proteins (Fig. 5F upper panel). The mouse monoclonal 5F2B3 recognizes the C-terminus of the CC domain of these proteins and also recognises the full-length proteins, whereas anti-NTD recognises full-length proteins and the N-terminus. 5F2B3 labelling also revealed a lower band of ∼ 60kDa indicating probable N-terminal degradation (Fig. 5F lower panel). This data led us to question the stability of mEFH1+2 and we therefore analyzed cells after 24 hours expression followed by a six-hour treatment with the proteosome inhibitor MG132 [26]. Cells were then probed with anti-NTD (S3A Fig.) and counted for positive mEFH1+2 signal (S3B Fig.). We also tested mEFH1+2 levels by western blotting using 5F2B3 (S3C Fig.).

The counts illustrated that 3.9% (*n* = 633, SE± 0.29%) of the population were positive for mEFH1+2 signal in the absence of MG132 treatment, whereas 23.3% (*n* = 559, SE± 3.5%) were positive after MG132 treatment (S3A, B Fig.). Quantification of western blots of mEFH1+2 probed with 5F2B3, and normalization with anti-tubulin loading control, indicate that there is 1.5 x fold more mEFH1+2 protein in cells after MG132 treatment (S3C Fig.). Oddly, we did not observe the ∼ 60kDa band noted previously in Fig. 5F in these untreated cells and are uncertain why, but it may signify that degradation of mEFH1+2 is not consistent and that degradation rates and origin on mEFH1+2 can vary.

Importantly, this data indicates that there is up to 40% degradation of mEFH1+2 when expressed in U-2OS cells, but more significantly no linear, helical or annular polymers were detected in cells in the absence or presence of MG132 treatment.

### BILBO1 truncations form polymers in trypanosomes

**Fig 6:**
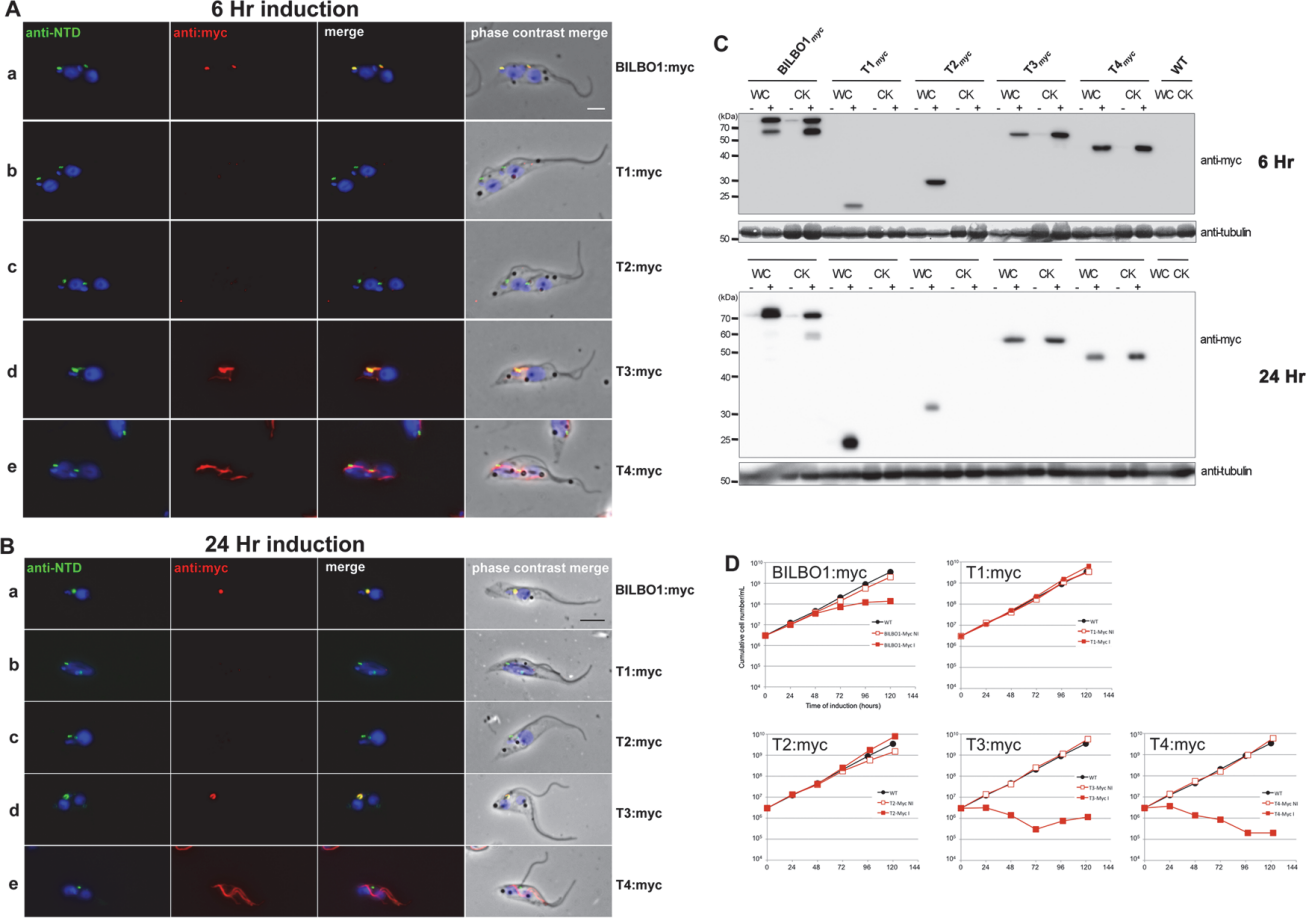
The coiled-domain is required for polymer formation in *T. brucei*. (A) Immunofluorescence labelling of cytoskeletons from cells expressing BILBO1:myc, T1:myc, T2:myc, T3:myc and T4:myc using anti-NTD (green) and anti-myc (red) after six hours of induction. (B) Immunofluorescence labelling of cytoskeletons expressing BILBO1:myc, T1:myc, T2:myc, T3:myc and T4:myc using anti-NTD (green) and anti-myc (red) after 24 hours of induction. (C) Western-blot of corresponding cells. 2.10^6^ non-induced (-) or induced (+) for six hours from whole cells (WC) or cytoskeletons (CK) were loaded on a 12% SDS-PAGE, transferred and immuno-probed with anti-myc, or anti-tubulin (tubulin is a loading control). WT is the non-transfected parental cell line. (D) Growth curves of the parental cell line (WT, black circles) compared to the cell lines non-induced (NI, red open squares) or induced (I, red closed squares) for the expression of BILBO1, T1, T2, T3, T4 myc-tagged truncations. Scale bars in A and B represent 5μm.

Our previous work has shown that a brief ectopic expression of a full-length tagged version of BILBO1 protein (GFP-BILBO1) in trypanosomes results in targeting and assembly into the FPC [8]. We accept the caveat that the expression of truncated, mutated or full-length forms of BILBO1 in trypanosomes will clearly involve the binding and/or interaction of other FPC proteins, which may influence the structures produced. However, we wanted to observe whether ectopic expression of these proteins would indeed form polymers in a trypanosome context. To avoid potential steric hindrance due to GFP and to discriminate between the endogenous BILBO1, we created C-terminus myc-tagged forms of the protein that are identical to those expressed in U-2 OS cells (for clarity, described here as T1:myc—T4:myc) and expressed them in procyclic *T*. *brucei* cells (Fig. 6), using the previously described tetracycline inducible expression system [8,27]. *T*. *brucei* cells expressing different truncations were detergent extracted to make cytoskeletons (CK) and simultaneously probed with the following antibodies anti-NTD (which labels the endogenous BILBO1 and BILBO1:myc, T1:myc, T2:myc, but not T3:myc or T4:myc truncations, in which case 5F2B3 was used), and anti-myc, which labels only myc tagged ectopic proteins.

After six hours of induction we observed BILBO1:myc at the FPC thus demonstrating that the myc tag does not affect the localization of the protein (Fig. 6A a). By western blotting we noted an additional band present under the main band (Fig. 6C) after six or 24 hours of expression and suggest that this is a degradation product. It is weaker after 24 hour expression compared to six hours suggesting more complete degradation to small peptides, much less degradation or degradation during or after sample preparation (for example when making cytoskeletons) (Fig. 6C).

T1:myc or T2:myc truncations were observed in the cytoplasm by WB (Fig. 6C), but no signal was present in detergent extracted cytoskeletons by immunofluorescence or WB (Fig. 6A b-c, 6B b-c, 6C). The solubility of T1:myc and T2:myc truncations is in agreement with the soluble forms observed when expressed in U-2 OS cells and, with respect to trypanosomes, it indicates that neither the N-terminus nor the EF-hand domains are sufficient for targeting or binding to the FPC. Extensive expression of T1:myc or T2:myc truncations (>24 hours) did not dramatically affect cell morphology or cell growth (Fig. 6B, and D) indicating that neither of these domains induce dominant negative effects. When probed with anti-NTD antibody endogenous BILBO1 localization at the FPC was not impaired or modified during T1:myc or T2:myc expression because the NTD-labelling was unmodified. By probing the samples with NTD, and an anti-tubulin loading control, on western blots we were able to measure the native levels of BILBO1 expression in cells expressing T1:myc or T2:myc truncations for six or 24hours and these were both shown to be 1.4 x higher than wild-type levels. After 24 hours induction these levels remained at 1.4 x higher than wild-type levels (S4 A Fig).

Immunofluorescence on procyclic trypanosome cytoskeletons and western blot analysis of whole cells (WC) or cytoskeletons (CK) demonstrated that T3:myc truncation is insoluble and is associated with the cytoskeleton (Fig. 6A d and 6B d). T3:myc targets primarily to the FPC, but also forms a subset of short fibres that below the FPC (Fig. 6A d). Surprisingly, these fibres were myc positive, but NTD negative, implying that they were formed predominately by T3:myc truncation. In contrast to T1:myc and T2:myc, longer expression of the T3:myc truncation resulted in targeting and binding to the FPC with deleterious effects; endogenous BILBO1 (labelled with anti-NTD) and T3:myc co-localized to a single FPC structure from which both flagella (old and new) emerged (Fig. 6B d).

Moreover, in these T3:myc induced cells, the new flagella were detached from the length of the cell body. This phenotype was characterised by flagellum attachment at the basal body region, but detachment along the length of the cell. For simplicity we have called this a “detached flagellum” phenotype. These cells died within 24 hours of T3:myc induction (Fig. 6D). Interestingly, detached flagella and cell death are phenotypes observed in the induced BILBO1 RNAi cell line [8].

Expression of T4:myc for six hours produced long fibre-like polymers that were observed within the cytoplasm (Fig. 6A, e). This implies that the CC domain, in the absence of the N-terminal domain or the EF-hand domains, is able to form polymers in *T*. *brucei* as well as in U-2 OS cells. Similarly to T3:myc, expression of >24 hours of T4:myc produced polymer structures that did not contain endogenous BILBO1 signal (Fig. 6B, e). Notably, cells expressing T4:myc died within 24 hours of induction (Fig. 6D) and, as with T3:myc expression, these cells had a detached new flagella phenotype (Fig. 6B e).

As with T1:myc and T2:myc, we probed the T3:myc and T4:myc protein samples by western blot with NTD, anti-myc and anti-tubulin and measured the native levels of BILBO1 expression. After 6 hours of induction, endogenous level of BILBO1 appeared to be 6.6 x fold and 5.1 x fold higher in T3:myc and T4:myc truncation expressing cells respectively. After 24 hours of expression these levels were shown to be 6.3 x and 4.2 x fold higher respectively compared to wild-type levels, (S4 B Fig.).

**Fig 7:**
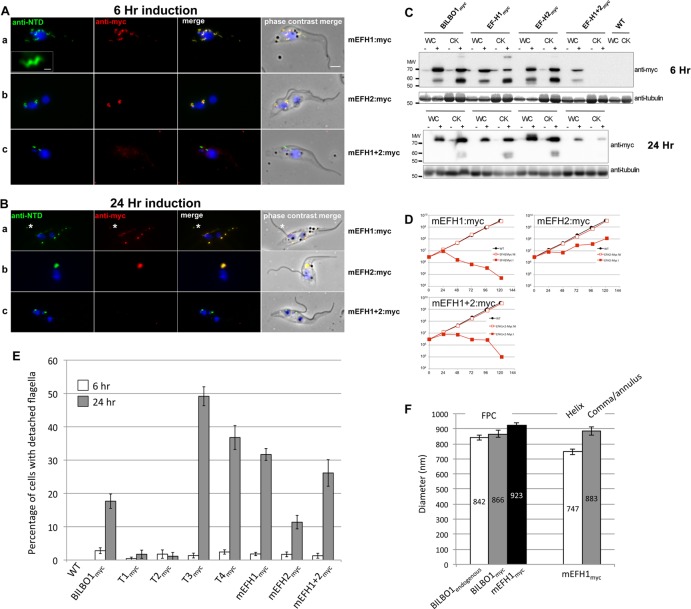
The EF-hand domains modulate the morphology of polymers formed by BILBO1. (A) Immunofluorescence labelling of cytoskeletons from cells expressing mEFH1:myc, mEFH2:myc, mEFH1+2:myc using anti-NTD (green) and anti-myc (red) after six hours of induction. Scale bar in the magnified inset represent 1 μm. Scale bar for all other images represents 5μm. (B) Immunofluorescence labelling of cytoskeleton extracted cells expressing mEFH1:myc, mEFH2:myc, mEFH1+2:myc using anti-NTD (green) and anti-myc (red) after 24 hours of induction. The asterisks indicate a dot in the new detached flagellum. (C) Western-blot of corresponding cells. 2.10^6^ cells (WC) or cytoskeletons (CK) non-induced (-) and induced (+) for six hours, were loaded on a 10% SDS-PAGE, transferred and immuno-probed with anti-myc and anti-tubulin as loading control. WT is the non-transfected parental cell line. All expressed proteins except mEFH1+2:myc are insoluble. (D) Growth curves of the parental cell line (WT, black) compared to the cell lines non-induced (NI, red open squares) or induced (I, red closed square) for the expression of mEFH1, mEFH2, mEFH1+2 myc-tagged proteins. (E) Overexpression of BILBO1 coiled-coil truncations or EF-hand domain mutations induces detached flagellum phenotypes. The percentage of cells with a detached flagellum was determined using phase contrast microscopy after six or 24 hours of protein expression. (F) WT cells or cells expressing recombinant proteins (induced for six hours), were fixed and immunolabelled with anti-myc and anti-NTD. The graph represents the measurements of the FPC diameter in WT cells, in BILBO1:myc, mEFH1:myc, mEFH2:myc and mEFH1+2:myc expressing cell lines. The graph also shows the diameter of the helix and comma/annulus shaped polymers formed by mEFH1:myc.

Given that the expression of T3:myc or T4:myc truncations in trypanosomes is lethal and induced detached flagellum phenotypes we counted the percentage of cells exhibited this phenotype after six or 24 hours of expression and compared this to T1:myc, T2:myc and BILBO1:myc expression. Fig. 7E shows that wild-type cells do not have a detached flagella phenotype, and cells expressing T1:myc and T2:myc truncations produced less than 2% detachment. However, after a 24 hour induction T3 cells showed a 36 x fold increase in detached flagella phenotypes, T4 cells showed 15.2 x fold increase and BILBO1:myc showed a 6.3 x fold increase (please refer to Table 4).

**Table 4:** T1:myc—T4:myc and BILBO1:myc detached flagellum phenotypes after expression *T. brucei* cells.

Time of induction	T1:myc detached flagella	T2:myc detached flagella	T3:myc detached flagella	T4:myc detached flagella	BILBO1:myc detached flagella
6h	**0.43%** (SE±, 043) *n* = 183	**1.77%** (SE± 1.25) *n* = 111	**1.35%** (SE± 0.72) *n* = 412	**2.41%** (SE± 0.65) *n* = 556	**2.8%** (SE± 0.87) *n* = 529
24h	**1.74%** (SE± 1.23) *n* = 125	**1.14%** (SE± 1.14) *n* = 97	**49.17%** (SE± 2.84) *n* = 228	**36.79%** (SE± 3.57) *n* = 406	**17.66%** (SE ±2.15) *n* = 495

Quantification of the percentage of cells exhibiting detached flagella phenotypes after six and 24 hours expression of T1:myc—T4:myc tagged proteins or BILBO1:myc tagged protein in procyclic *T*. *brucei* cells. T3:myc, T4:myc and BILBO1:myc induced detached flagella phenotypes whereas T1:myc and T2:myc did not.

This data suggest that the high number of detached flagella phenotypes induced by BILBO1:myc, T3:myc and T4:myc expression is likely to be a dominant negative effect and the secondary effects of this have influenced the formation or function of the flagella attachment zone (FAZ) [6] resulting in detached flagella. The results obtained by Y2H analysis for T1-T4 correspond with the results obtained using the same truncations expressed in U-2 OS cells and in trypanosomes. Essentially, For T1 and T2 there is no BILBO1 interaction by Y2H, also no polymer formation was observed when these truncations were expressed, but positive protein-protein interaction and polymer formation was observed with expression of full-length BILBO1 or T3 or T4 truncations. These data indicate that the CC domain is required for polymerization and targeting/binding to the FPC. It also illustrates that over-expression (24 hours) of the CC domain alone (T4:myc) is sufficient to induce the detached flagellum phenotypes.

### EF-hand 1 domain influences BILBO1 polymer shape in *T*. *brucei*

To analyse in detail the role of the BILBO1 EF-hand domains in trypanosomes we expressed mEFH1:myc, mEFH2:myc, and mEFH1+2:myc in *T*. *brucei* procyclic cells (Fig. 7). Expression of mEFH1:myc for six hours demonstrated targeting to the FPC, but in addition we observed the formation of several helical structures independent of the FPC (Fig. 7A a). The diameter of the helices and comma/annuli structures after six hours of expression was 747 nm (*n* = 67, SE± 20nm) and 883 nm (*n* = 39, SE± 27nm) respectively (Fig. 7F).

As with T1—T4 experiments, we probed the proteins from cells expressing BILBO1:myc and mutated EF-hand:myc by western blot and compared them to the native levels of BILBO1. Notably, we observed degradation bands at ∼ 60kDa, which is probably due to the same degradation related reasons we have specified earlier for the expression of BILBO1:myc (Fig. 6A a). Nevertheless, after six hours of expressing BILBO1:myc, mEFH1:myc, mEFH2:myc, or mEFH1+2:myc, wild-type BILBO1 protein levels were shown to be 1.1 x, 0.9 x 1.3 x and 0.9 x fold higher or lower respectively than the expressed myc tagged levels, (S4, C Fig.). After 24 hours of expression these levels were 2.5 x, 1.3 x, 2.9 x, and 1.0 x higher respectively than myc tagged protein levels.

Because mEFH1:myc formed helices when expressed in trypanosomes, we wanted to compare helix dimensions with that of the FPC. We therefore measured the diameter of the FPC (major axis diameter) in wild-type (endogenous BILBO1), BILBO1:myc and mEFH1-myc expressing cells (Fig. 7F). We measured the FPC diameter of wild type G1 procyclic cells after immunolabelling with anti-NTD antibody and the FPC of BILBO1:myc and mEFH1-Myc expressing cells, for six hours, using anti-myc antibody. The diameter of wild-type FPC was 842 nm (as noted in Table 3) whilst in the BILBO1:myc expressing cells the diameter was measured as 866 nm (*n* = 48, SE± 20nm) and was 923 nm (*n* = 88, SE± 20nm) in mEFH1:myc expressing cells (Fig. 7F).

The fact that mEFH1:myc structures were helical indicates that mutation of EF-hand domain 1 influences the type of polymer formed, in the context of the trypanosome (Fig. 7A a). Expression of mEFH1:myc (>24 hours) induced the formation of myc and NTD positive, globular, insoluble structures followed by cell death occurring between 24–48 hours of induction (Fig. 7B, D). Noticeably, as previously observed in T3 and T4 expressing cells, and in BILBO1 RNAi knockdown cells [4,8], expression of mEFH1:myc induced detached flagella phenotypes (Fig. 7B a) (see below for quantification). We observed the formation of a single insoluble BILBO1 positive structure within the length of the new detached flagellum. The presence of this structure along the flagellum suggests the mis-targeting of this mEFH1:myc form of BILBO1 (Fig. 7B a, asterisk).

Similar to observations made with BILBO1:myc, the mEFH2:myc targets to the FPC after a six hour induction, suggesting that EF-hand domain 2 function is not required for targeting to the FPC (Fig. 7A b). Expression >24 hours resulted in cells with new flagella detached phenotypes (see below for counts) and the formation of large insoluble structures that were not associated with the old or new flagella. Cell death occurred between 24 and 48 hours (Fig. 7B b and 7 D).

The majority of mEFH1+2:myc protein when expressed in trypanosomes for six hours was soluble, as observed by WB (Fig. 7C). As in the expression of mEFH1+2 in U-2 OS cells we cannot rule out the possibility of rapid degradation since degradation bands were observed after six hours, and to a much lesser extent 24 hours, of expression. Indeed mEFH1+2:myc expressing cells treated with MG132 had 2.2 x fold higher protein levels when compared to non-treated cells suggesting mEFH1+2:myc degradation *via* the proteasome (S4D Fig.). A weak mEFH1+2:myc signal could be detected on cytoskeletons probed by immunofluorescence and western-blot (Fig. 7A, B, C) and extensive expression of mEFH1+2:myc (>24 hours) resulted in a very weak mEFH1+2:myc immunofluorescence signal close to both old and new FPC structures. When probed by WB a weak signal was also observed in cytoskeleton samples that had been expressing mEFH1+2:myc for 24 hours (Fig. 7 C). Cells died after >24 hours expression of mEFH1+2:myc (Fig. 7B, D). Long induction also induced the production detached flagella phenotypes (see Table 5 for quantification). In all cases where mutated mEF-hand proteins were expressed we noticed that after 24 hours of expression little or no anti-BILBO1 signal (endogenous or myc-tagged) was detected at the base of the new flagellum suggesting sequestration or degradation of wild-type BILBO1 (Fig. 7B a-c).

Since the expression of mEFH1:myc, mEFH2:myc or mEFH1+2:myc in trypanosomes induced detached flagella phenotypes, we investigated the extent of their appearance. We observed a significant difference between a six hour expression and 24h expression of mEFH1:myc because the number of detached flagella phenotypes increased by 17 x fold. For mEFH2:myc expression we observed a 6 x fold increase in detached flagella and for mEFH1+2:myc there was a 20 x fold increase in detached flagella phenotypes, (please refer to Table 5 and Fig. 7E).

**Table 5:** mEFH:myc induced detached flagellum phenotypes after expression in *T*. *brucei* cells.

Time of induction	mEFH1:myc detached flagella	mEFH2:myc detached flagella	mEFH1+2:myc detached flagella
6h	**1.83%** (SE± 0.49) *n* = 852	**1.7%** (SE± 0.71 *n* = 489	**1.29%** (SE± 0.74) *n* = 181
24h	**31.7%** (SE± 1.76) *n* = 502	**11.37%** (SE± 2.02 *n* = 338	**26.14%** (SE± 3.98) *n* = 148

Quantification of the percentage of cells exhibiting detached flagella phenotypes after six and 24 h expression of mEFH1:myc, mEFH2:myc and mEFH1+2:myc tagged proteins in procyclic *T*. *brucei* cells. Expression of all EF-hand mutations induced detached flagella phenotypes.

## Discussion

The results in this study support the concept that BILBO1 has self-assembly properties that are dependent on the CC region of the protein, and that can be influenced by the EF-hand domains at its N-terminus. We hypothesize that BILBO1 is well suited to play a role in the formation of the FPC annulus. The results reported here establish that BILBO1 can, autonomously, support the formation of various polymers including helical polymers. We suggest that these polymer-forming properties are important in building a FPC.

We demonstrate here that the two tandem EF-hand domains of BILBO1 bind calcium *in vitro* and that mutation of these domains either together or independently prevented calcium binding. The observation that mutation of one EF-hand domain prevented the second from binding calcium is not unprecedented. In fact, the basic unit for a functional EF-hand protein is a pair of EF-hand domains that stably form a four-helix bundle [28,29,30]. Previous studies have also shown that the four-helix bundle should be treated as a single global structure because the two EF-hand domains bind calcium cooperatively [30]. Indeed, our ITC titration fit curve for wild-type *Tb*BILBO1-EF-hand domains indicate that the molar ratio between Ca^2+^ and the protein was approximately 2 (Fig. 2E), suggesting that both EF-hand domains bind calcium. The expression in mammalian cells or trypanosomes of full-length BILBO1 with mutations on either or both EF-hand domains produced different types of polymers (see below), which would also suggest that both hands bind calcium.

The data obtained from Y2H analysis, U-2 OS and trypanosome studies strongly indicate that BILBO1 x BILBO1 interactions exist, and in many cases, are targeted to the FPC of trypanosomes. We have shown that long-term over-expression of full-length BILBO1:myc in *T*. *brucei* procyclic cells is lethal.

When expressed in *T*. *brucei* procyclic cells the T1 and T2 truncated forms of BILBO1 are soluble, but neither can interact with full-length BILBO1 nor with the CC domain, and do not form polymers. We can thus consider that the N-terminal domain of BILBO1 is not directly involved in BILBO1 polymerization. On the other hand, the N-terminal domain can regulate the shape of the polymer.

Expression of mutated EF-hand proteins in trypanosomes did not prevent targeting to the FPC indicating that they bind to native BILBO1. mEF-hand 1+2 location to the FPC was very weak when detected by immunofluorescence, but it was also mostly degraded under these conditions. Further, we carried out yeast two-hybrid analysis to test whether full-length BILBO1 interacts with full-length BILBO1, or deltaEF-H1+2 (a deleted EF-hand form of BILBO1 where the N-terminal domain is retained) and full-length BILBO1 versus mEF-hand1+2. We also tested mEF-hand1+2 versus T4 truncation, or mEF-hand1+2 versus T3 truncation. In all cases BILBO1 interacted with these modified proteins indicating that the EF-hand domains are not required for this interaction. Interestingly, the double EF-hand mutant mEF-hand1+2 also bound to BILBO1 indicating that neither lack of calcium binding nor the conformational change induced by calcium binding prevents BILBO1-BILBO1 interactions (S5 A Fig.).

Recently, nuclear magnetic resonance (NMR) and X-ray crystallographic techniques were used to solve the 3D structure of the N-terminal domain of BILBO1. That work involved experiments demonstrating that the N-terminal domain has an exposed surface patch within an ubiquitin-like fold. Expression in trypanosomes of full-length-BILBO1 mutated on this patch is lethal suggesting that this region has important functional roles which may include interaction with other FPC proteins [14]. Additionally, the authors also showed that EF-hand domains of BILBO1 changes its conformation upon calcium binding, and the CC domain forms anti-parallel dimers and linear polymers and these filaments can condense into fibres through lateral interactions [15].

When expressed in *T*. *brucei* procyclic cells, T3:myc and T4:myc fragments localised to the FPC, and produced lethal effects. It is also possible that they induced dominant negative effects because detached flagella phenotypes were observed after expression of these proteins, indicating a probable interruption of flagellum attachment zone (FAZ) function or biogenesis.

When expressed in U-2 OS cells, T1 and T2 truncation were soluble, and T3 and T4 truncations formed polymers. Interestingly, expression of the T4 truncation formed exclusively linear spindle-like polymers in these cells. From these results we conclude that the CC domain of BILBO1 can spontaneously form homo-polymers *in vivo* and these are linear due to the absence of the N-terminus, which functions to modulate the type of polymer formed. It is not clear how BILBO1 polymers are formed in U-2 OS cells and although trypanosome FPC proteins are absent there may be other proteins interacting with BILBO1 contributing to the formation of polymers.

Since the FPC has been established in earlier published studies to be an annulus/horseshoe *in vivo* we suggest that, in the parasite, BILBO1 could form the template for the FPC, which then associates with other FPC proteins perhaps under the modulation of calcium to form the final annulus/horseshoe [4,8]. Further evidence for BILBO1 calcium binding and polymer forming properties was provided by Vidilaseris *et al.*, 2014 [15]. Using *in vitro* structural dissections of BILBO1 they showed that the EF-hand domains change conformation upon calcium binding, and the central CC can form anti-parallel dimers. They demonstrated that a C-terminal leucine zipper is present and appears to contain targeting information so that inter-dimer interactions can form between adjacent leucine zippers of the CC domain, which allow BILBO1 to form extended filaments. Finally, they showed that these filaments could condense into fibers through lateral interactions [15]. From these observations it is suggested that two BILBO1 molecules can form an anti-parallel dimer *via* their CC domains, which assemble into a filament through the interactions between the C-terminal leucine zippers. BILBO1 forms polymers spontaneously *in vitro* with no apparent need for nucleotide hydrolysis [15], but spontaneous polymerization is not unprecedented for cytoskeleton proteins and has been observed for intermediate filaments and bacterial flagellins [31,32,33,34].

Within the parasite BILBO1 is unlikely to function alone and must interact with other proteins to form the scaffold of the FPC. Based on Y2H analysis we have identified numerous BILBO1 binding proteins, two of which bind to the N-terminus of BILBO1. One of these proteins, FPC5, is kinetoplastid specific, but it is feasible that other BILBO1 binders are structural and may influence BILBO1 conformation *in vivo*. Indeed it is also possible there is interplay between calcium and protein binding to BILBO1 and this may regulate how BILBO1 functions or perhaps even form polymers. Data to support this in presented in S5B Fig. where we use Y2H assays to illustrate that the BILBO1 binding domain of FPC5 interacts with full-length BILBO1 as bait or prey, but does not interact with BILBO1 if the EF-hand domains have been deleted or mutated to prevent calcium binding.

In order to test the effects of calcium chelation on polymers we treated U-2 OS cells, which have expressed untagged BILBO1 for six or 24 hours, with the membrane permeable calcium chelator BAPTA-AM. We did not observe any difference between treated and untreated cells (S6A Fig.) suggesting that once polymerization occurs the protein is somewhat stable to calcium chelation. We also treated cytoskeletons derived from cells that had expressed BILBO1myc or the EF-hand mutants, with 50mM EGTA, but the chelation treatment did not disrupt the FPC nor solubilize the polymers formed by mEF-hand 1 domains under these conditions (S6B Fig.). The former results suggests that these polymers may have very strong and/or irreversible inter-molecule interactions, and the latter result is probably due to both strong interactions and the stabilizing properties of other FPC proteins that prevent depolymerisation. Such stabilization is not unprecedented and a good example of this is alphaB-crystallin, which has been demonstrated to stabilize microtubules against calcium depolymerization [35,36].

The results observed from the ITC studies and the effects of the mutation of the EF-hand domains in BILBO1 on polymer assembly *in vivo* demonstrates that BILBO1 does binds calcium. The expression of mEFH1 in U-2 OS cells produced small aggregates, whereas expression of mEFH2 in these cells induced the formation of helical polymers. When expressed in trypanosomes mEFH1 formed helical polymers whereas mEFH2 did not. We do not have a clear explanation for this difference between mammalian cells and trypanosomes, but one can speculate that it may reflect a change in three-dimensional structure when the protein in question interacts with, or binds to, parasite specific proteins. It is apparent from our studies and confirmed by other work that mutating calcium-binding domains can also influence BILBO1 structure and this may influence if and how polymers are formed [15]. The fact that BILBO1 can form different shapes such a linear or annular/comma shaped polymers is especially interesting when we consider the helices formed when either EF-hand domain was mutated. Therefore it is possible that these mutations prevent calcium binding and could stop the domain folding correctly, which could result in alternative BILBO1 polymerization.

In trypanosome procyclic cells mEFH1:myc targeted to the FPC, but also within the new detached flagellum of these cells. It is unclear why these mis-targeted mEFH1 “spots” are present within the length of the flagellum, but it may be due to the limited ability to bind to partners for FPC retention or may be due to modified access to the intraflagellar transport system. Mutated EFH1+2:myc was apparently predominantly soluble when expressed in trypanosomes, but in this context it is not unusual, because the mutation of a single amino acid has been noted in some cases to modify protein solubility such as maltose binding protein and haemoglobin [37,38,39]. We also show that EFH1+2:myc is mostly soluble, but can form some small punctate or aggregates in U-2 OS cells and is predominantly degraded when expressed. Longer expression of EFH1+2:myc in trypanosomes induced flagella detachment phenoptypes suggesting perturbation of the FAZ. Low levels appear to be very toxic to cells perhaps by recruiting inappropriate proteins or preventing the correct binding of partner proteins such as FPC5.

In the trypanosome expression experiments the mutant mEF-hand 1 and 2 proteins are only recognized by the anti-myc antibody and show that they do target to the FPC. This provides some evidence to suggest that in a six-hour induction they do not sequester all native BILBO1 from targeting to the collar. After 24 hours of expression this is not the case and native BILBO1 does appear to be degraded, not targeted to, and/or sequestered from the FPC as seen in Fig. 7, B a and b. The sequestering of wild-type BILBO1 may also be the cause of flagella detachment and cell death.

The diameter of mEFH2-induced helices in U-2 OS cells was 730 nm and comma diameter was 787 nm whilst annuli diameter was 771 nm. The diameter of the helices formed by procyclic cells expressing mEFH1:myc is 750nm, whilst the diameter of the wild-type FPC was 842 nm. The similarity between the diameters of these polymers compared to the FPC is probably not coincidental and could be envisaged to be associated with the intrinsic properties of BILBO1. Based on our observations, we propose a model in which BILBO1 is a structural scaffold of the FPC and assembles into polymers *via* the CC domain and leucine zipper as proposed by Vidilaseris, *et al.*, 2014 [15]. This polymer forms an elliptical or annular structure, the exact state depending on the calcium-binding functionality of the EF-hand domain and the presence or absence of partner proteins. In this model the role of the EF-hand domain is vital and is involved in the plasticity of the structure, but clearly in the trypanosome there must be other proteins that bind to or regulate the structure of the FPC. If we consider a hypothesis where the FPC changes dimensions to accommodate a new flagellum during the cell cycle then one hypothetical interpretation could be that the interplay with binding partners is important during the parasite cell cycle, wherein an annular/horseshoe shaped FPC is required in early cell cycle stages, whilst a more elliptical FPC is required during emergence of the new flagellum during S/G2 or later. Nevertheless, it is unclear how precisely a new FPC is formed. If a new FPC is formed *de novo* then we would expect much less need for dramatic changes in its shape and this interpretation provides a role for BILBO1 whereby it forms a FPC as the new flagellum exits the FP.

The primary and motile cilia and the flagella of differentiating spermatids have a ciliary pocket (CP) [40,41,42], which is physically associated with clathrin-coated endocytotic vesicles [40,41] and shares a conspicuously similar structure to the FP. Surprisingly, it is not known if the equivalent of the FPC, the CP collar (CPC) actually exists. Therefore it is unknown if the CP indeed requires a BILBO1-like protein or if the CP is constructed differently to the FP.

The importance of a precisely constructed primary cilia cytoskeleton is revealed by studies on ciliopathies; defective primary cilia in humans lead to polycystic kidney disease and a variety of other illnesses [43,44,45,46,47]. Clearly, a thorough understanding of how the FP and/or the CP are formed will provide important insights into both parasite biology and human ciliopathies. The data we have reported here have elucidated *in vivo*, and in a tractable system, some interesting properties of BILBO1 and these have advanced our understanding of how the FP is constructed. The ongoing search for the identification and characterization of additional FPC proteins will add to our understanding of the ways in which the FPC is organized and maintained. We anticipate that this data will be useful to obtain a more general understanding of the assembly of kinetoplastid FPC complexes and provide important clues on how to inhibit FPC biogenesis.

## Materials and Methods

### Cell lines, cell culture and cell transfection

U-2 OS cells (human bone osteosarcoma epithelial cells, ATCC Number: HTB-96 [48] were grown in D-MEM Glutamax (Gibco) supplemented with final concentrations of 10% fetal calf serum (Invitrogen), 100 units.mL^-1^ of Penicillin (Invitrogen), and 100 μg.mL^-1^ of Streptomycin (Invitrogen) at 37°C plus 5% CO_2_. Exponentially growing U-2 OS cells in 24 well plate with glass coverslips were lipotransfected as in Dacheux et al., [49] with 0.5–2 μg DNA using Lipofectamine 2000 in OPTIMEM (Invitrogen) according to the manufacturer’s instructions and processed for IF six to 24 hours post-transfection.

The *BILBO1* ORF and truncations were amplified by PCR from *T*. *brucei* TREU927/4 GUTat10.1 genomic DNA [50]. The work described in this study uses the parental procyclic form (PCF) *T*. *brucei* 427 29–13 cell-line, co-expressing the T7 RNA polymerase and tetracycline repressor, named for the purposes of this study as wild-type (WT) [51]. WT cells were transfected with NotI linearized plasmids as in [52] and cloned. Expression of recombinant proteins was induced with 1 μg.mL^-1^ tetracycline. Growth curves were done by using a mallassez cell counter every 24 hours and by diluting the cells back to 3.10^6^ cells/ml. Growth curves in Figs. 6 and 7 represent the cumulative cell number.

### Vectors

**Mammalian expression vectors.** The *BILBO1* ORF and truncations were cloned into the pcDNA3 between HindIII-XbaI sites for *BILBO1* full length and EcoRI-XhoI for the truncations or into pcDNA3.1 CT-GFP TOPO (Invitrogen). Mutations of the EF-hand domain 1 (D194A (GAT/GcT); N198A (AAC/gcC); D202A (GAC/GcC); D205A (GAC/GcC), the EF-hand domain 2 (D230A (GAC/GcC); N232A (AAC/gcC); E241A (GAA/GcA), and the serine 163 mutations (S163D (TCG/gat) and S163A (TCG/gCG) were done by site-directed mutagenesis following the instructions from the Agilent QuickChange Site-directed Mutagenesis kit.

**Trypanosome expression vector.** The pLew100X-3myc has been modified in the laboratory from pLew100 [49]. BILBO1 and truncations 1, 2, 3, 4, and mutated EF-hands versions of BILBO1 were cloned into pLew100X-3myc between the *HindIII-XbaI* sites.

**Yeast two-hybrid vectors.** Open reading frames were amplified by PCR from *T*. *brucei* PCF genomic DNA and cloned in the prey (pGADT7-AD, Clontech) and bait (pGBKT7, Clontech) vectors between the *EcoRI-BamHI* sites.

### Immunofluorescence

**In trypanosomes.** For cytoskeleton preparations, cells were washed in PBS, loaded on poly-l-lysine coated glass slides, and extracted with 1% or 0.25% NP40 in Pipes buffer (100 mM Pipes pH6.9, 1 mM MgCl_2_) for 5 minutes then washed twice in Pipes buffer. Cytoskeletons were fixed in -20°C methanol or 3% paraformaldehyde (PFA) in PBS. After PFA fixation, cells were neutralized 10 min in glycine (100 mM in PBS). After 3 washes in PBS, samples were incubated with the primary antibodies for 1 hour at room temperature in a moist chamber: anti-BILBO1 (mouse monoclonal 5F2B3, which recognizes the CC domain [8], 1:10 dilution) or rabbit anti-NTD (which recognises the first 110 aa of BILBO1, [9]) diluted 1:50 in PBS. After two PBS washes, cells were incubated for 1 hour with the secondary antibodies anti-mouse-IgG (H+L) conjugated to Alexa 594 (Molecular Probes A21201, 1:400 dilution) or FITC (Sigma F-2012, 1:100 dilution), or anti-rabbit IgG (H+L) from goat conjugated to FITC (Sigma #F-9887, 1:100 dilution). The nuclei and kinetoplasts were labeled with DAPI (10 μg.mL^-1^ in PBS for 5 minutes), washed twice in PBS for 5 minutes. Slides were mounted with Slowfade Gold (Molecular Probes S-36936).

**In U-2 OS cells.** For observation of whole cells, transfected U-2 OS cells were fixed in 3% PFA in PBS for 15 minutes (at RT or at 37°C). When indicated, transfected cells were incubated with the membrane permeable calcium chelator BAPTA-AM (Sigma A1076, 25μg/ml final concentration) for three hours or with the proteasome inhibitor MG132 (Sigma C2211, 20–50 μM final concentration) for six hours before fixation. To remove soluble proteins, cells were briefly extracted for 2 min with 30 μl of EMT, TX-100 0.5%, glycerol 10% then fixed in PFA 3% (at 37°C, 15 min). After fixation, cells were neutralized 10 min in glycine (100 mM in PBS). After two washes in PBS, cells were incubated in permeabilization buffer PB (PBS, 10% foetal calf serum, 0.1% saponin) for 10–30 minutes. Primary antibodies anti-BILBO1 (mouse monoclonal 5F2B3), [8] 1:10 dilution, anti-NTD BILBO1 (which binds to aa 1–110, rabbit polyclonal, 1:50 dilution, [9]), anti-alpha-tubulin DM1A (Sigma T9026, 1:500 dilution) or TAT1 (1:100 dilution, [53]), anti-calnexin (rabbit polyclonal, 1:500 dilution), anti-giantin (rabbit polyclonal, 1:750 dilution), anti-Vimentin V9 (Interchim NB200-622, 1:250 dilution) were added and the slides were incubated for 1 hour in a dark, moist chamber. After two PBS washes, cells were incubated for 1 hour with the secondary antibodies anti-mouse-IgG (H+L) conjugated to Alexa-594 (Molecular Probes A21201, 1:400 dilution), or to FITC (Sigma F-2012, 1:100–1:400 dilution), or to anti-rabbit Texas-Red-conjugated (Molecular Probes T-6391, 1:400 dilution), or to anti-rabbit FITC-conjugated (Sigma F-9887, 1:100–1:400 dilution). For the F-actin labelling, Texas-red-conjugated phalloidin (Molecular Probes A12380, 1:160 dilution) was incubated with the secondary antibody. The nuclei were stained with DAPI (0.25 μg.mL^-1^ in PBS for 5 minutes) and cells were washed and mounted with Prolong (Molecular Probes S-36930).

Images were acquired on a Zeiss Axioplan2 or a Zeiss Imager Z1 microscope, using a Photometrics Coolsnap HQ2 camera, with Zeiss 100x or 63x objectives (NA 1.4) using Metamorph software (Molecular Devices), and processed with ImageJ. Polymer dimensions were measured using ImageJ. Total fluorescence intensities in U-2 OS cells were quantified from Z-stack acquisitions and using ImageJ on SUM intensity Z project, after background subtraction, and selection of each cell as region of interest. The measurement of polymers was done using fluorescence or immunofluorescence based images. The dimensions were measured from at least three separate experiments and were measured by hand using Image J software.

**Protein expression and purification.** *Tb*BILBO1 wild-type and mutated EF-hands proteins (residues 177–250; WT, mEFH1, mEFH2, and mEFH1+2), were cloned into the custom vector MalpET as described previously [15]. All recombinant proteins, each carrying an N-terminal MBP-His_10_ tag, were expressed in *E*. *coli* BL21 (DE3). Bacteria transformed with the cloned constructs were grown at 37°C to an A_600_ of ∼0.6–0.8 and then subjected to cold shock (ice, 30 min). Protein expression was induced by addition of 0.25 mM isopropylthio-β-D-galactoside, and protein production was continued for 20–22 hours at 16°C.

Cells were harvested by centrifugation (4,000×g, 20 min) and resuspended in cold lysis buffer (20 mM Tris-HCl pH 8.0, 300 mM NaCl, 20 mM imidazole, 5% (v/v) glycerol). The cells were broken open with an EmulsiFlex-C3 homogenizer (Avestin) and the lysate was cleared by centrifugation (16,000×g, 45 min; 4°C) to remove cell debris. The supernatant was filtered (0.45-μm pore size) and loaded onto a Ni-HiTrap column (GE Healthcare) pre-equilibrated with the same lysis buffer in order to capture the expressed proteins. The column was washed with 5 × column volume of lysis buffer, and bound protein was eluted by a linear gradient concentration of imidazole (20–600 mM, 10× column volume) in the lysis buffer. Target proteins were further purified on a Superdex S-200 16/60 column (GE Healthcare) pre-equilibrated with 20 mM Tris-HCl pH 8.0, 100 mM NaCl, 5mM DTT and 5% (v/v) glycerol. Fractions containing target proteins were pooled and concentrated according to requirements for subsequent experiments.

**Isothermal titration calorimetry (ITC).** For all ITC experiments, ultrapure water (milli-Q apparatus, Millipore) was used. All plastic materials were washed with 1 mM EDTA (pH 8.0) and then rinsed with milli-Q water to minimize Ca^2+^ contamination. ITC measurements were carried out using an iTC200 microcalorimeter (MicroCal) at 25°C in ITC buffer (20 mM Tris-HCl pH 8.0, 100 mM NaCl). Potentially pre-bound calcium was removed from the *Tb*BILBO1-EFh by incubating the protein with 50 mM EDTA (pH 8.0) for 1 hour at RT. EDTA was subsequently removed by dialyzing the protein sample against 3L of ITC buffer 5 times over 36 hours at 4°C. Before each ITC experiment, the sample cell of the microcalorimeter was washed several times with 1 mM EDTA (pH 8.0) and then rinsed with milli-Q water.

The sample cell was loaded with 200 μl of 30μM protein in ITC buffer. The reference cell contained only milli-Q water. Titration was carried out using a 40-μl syringe filled with 600μM CaCl_2_ prepared in ITC buffer under continuous stirring at 1,000 × rpm. Injections were started after baseline stabilization. Each titration experiment consisted of an initial 0.4-μl injection followed by 19 consecutive injections of 2 μl each with duration of 0.8 s. The interval between each two injections was 150 seconds. The heat of dilution was measured by injecting CaCl_2_ into the sample buffer without protein. The enthalpy change for each injection was calculated by integrating the area under the peaks for the recorded time course of power change, and then subtracting the control titration. Data were analyzed using the MicroCal Origin software and fitted to obtain thermodynamic parameters of calcium binding to the protein using a model with one set of sites.

**Electron microscopy.** Transfected U-2OS cells were harvested by scraping and then pelleted at 800 x g for 10 min at room temperature. They were then resuspended in 250μL PBS (plus protease inhibitors), for 30 min at 4°C to depolymerize the sub-pellicular microtubules. 10μL of cell suspension was placed on freshly charged formvar/carbon coated G200 nickel electron EM grids at (4°C). After the cells had adhered the grids were inverted onto extraction buffer (500μL PBS, 1% Nonidet P40 plus benzonase and protease inhibitors) and extracted for 15 minutes in at R/T. Grids were then washed (1 x 5 minutes) by floating on 500μL PBS and fixed 5 minutes in 500μL of 2.5% glutaraldehyde in PBS. Grids were then washed in 500μL water, 1 x 5 minutes and negatively stained with a 10μL drop/grid of 50:50 mix of NanoVan:NanoW. For striation measurements, digital images were taken from grids of at least three different experiments. Filaments were measured and striations were counted by hand on all filaments identified using Image J software.

### Yeast two-hybrid interaction assays

The pGADT7-AD (prey) and pGBKT7 (bait) based plasmid constructs were transformed in the yeast cell lines Y187 and Y2HGold respectively. After production of diploids cells, interaction tests were done using the drop test technique according to the manufacturer’s instructions (Matchmaker Gold Yeast Two-Hybrid System, Clontech). Haploid and diploid strains were grown in SC medium (YNB (w/o ammonium sulfate 1.7 g.L^-1^ (BD, #233520), Ammonium sulfate 5 g.L^-1^ (Euromedex, #2019), CSM (-Leu, -His, -Trp, -Ade, -Ura) 0.59 g.L^-1^ (MP, #4550–122), Dextrose (D+Glucose) 0.59 g.L^-1^ (Euromedex, #UG3050), Uracil (0.02 g.L^-1^) and complemented with Leucine (1 g.L^-1^), Tryptophan (0.05 g.L^-1^), Histidine (0.02 g.L^-1^), or Adenine (0.04 g.L^-1^) as required. Absence of auto-activation for each pGBKT7 bait construct was tested on SC-Tryptophan-Histidine medium. Absence of toxicity for each pGADT7-AD and pGBKT7 construct was tested on SC-Leucine and SC-Tryptophan respectively. Diploid yeasts were selected on SC-Leucine-Tryptophan medium (SC-L-W). Interaction tests were done on SC-L-W-Histidine media. All interactions were tested in both prey and bait configuration. Interaction using T2 as bait could not be tested because of auto-activation on SC-W-H medium.

### Bioinformatics

The two EF-hand domains were predicted by InterProScan [54] and Smart [55] software, and the CC domain by the Coils software [56].

### Sample preparation and western blots

**Trypanosome cells.** 2.5.10^7^ non-induced and induced cells (six or 24 hours) PCF were split in two flasks for whole cells (WC) and cytoskeleton (CK) samples. For WC samples, cells were spun at 1,000 x g for 10 minutes, washed once and resuspended at 1.10^6^ cells/μL^-1^ in PBS. An equivalent volume of 2x sample buffer and 25U of benzonase (Sigma, E1014) was added before boiling 3 minutes. For CK samples, cells were spun at 1,000 g for 10 minutes and washed once in PBS, EDTA 10mM and resuspended at 1.10^6^ cells/μL^-1^ in 100 mM PIPES pH6.8, 2 mM MgCl_2_, 0.25% NP-40, Protease inhibitor (Calbiochem, 1:10,000 dilution) and 25U of benzonase. After 10 minutes incubation on ice, cytoskeletons were pelleted at 1,000 x g for 30 minutes then washed in 1 mL 100 mM PIPES pH6.8, 2 mM MgCl_2_ and resuspended in the same buffer (1.10^6^ cells/μL^-1^ final). An equivalent volume of 2x sample buffer was added before boiling for 3 minutes. 2.10^6^ cells (or cytoskeleton) were loaded on 10 or 12% SDS-PAGE, semi-dry transferred on PVDF membrane or nitrocellulose membrane.

**U-2 OS cells.** Exponentially growing U-2 OS cells in T-25 flask were lipotransfected with 12.5 μg DNA using Lipofectamine 2000 in OPTIMEM (Invitrogen) according to the manufacturer’s instructions and processed for western-blot for 6 hours post-transfection (or 24 hours post-transfection for the mEFH1+2 sample). Cells were collected by; scraping the bottom of the respective culture flasks and transferring the detached cells into ice cold PBS. Cells were then centrifuged 5 min, 1,000 x g and resuspended in 125μL of buffer (PIPES 60 mM, HEPES 25 mM, EGTA 10 mM, MgCl_2_ 10 mM adjusted to pH6.9 with KOH, glycerol 10%, protease inhibitors (Calbiochem Cocktail set III, 1:10,000 dilution and 1mM PMSF) then lysed by adding 125μL of buffer supplemented with 0.2% TX-100. The supernatant was collected after a 5 minute centrifugation at 1,500 x g and 62.5μL of sample buffer 4x was added. Boiling 5 for minutes denatured the sample and then benzonase (5U) was added. The pellet was resuspended in 250 μL of buffer with 0.1% TX-100, 62.5μL sample buffer 4x was added and the sample was denatured by boiling for 5 minute before adding 7.5U of benzonase. Protein concentrations were assayed using the Pierce 660 nm Protein Assay (#22660) with the ionic detergent compatibility reagent (#22663) kit according to the manufacturer’s instructions. 13–20μg of protein from supernatant samples, and a corresponding volume of pellet samples, were separated by SDS-PAGE (10%) and semi-dry transferred onto PVDF membrane. Membranes were blocked in Tris-buffered saline (TBS), 0.2% Tween-20, 5% skimmed milk powder for 1 hour then incubated overnight at 4°C with the primary antibodies diluted in blocking solution: rabbit polyclonal anti-NTD diluted at 1:200, mouse monoclonal 5F2B3 undiluted, anti-myc monoclonal 9E10 (A kind gift from K. Ersfeld, University of Bayreuth, Germany) at 1:200, anti-alpha-Tubulin TAT1 monoclonal antibody (a kind gift from K. Gull, Sir William Dunn School of Pathology, University of Oxford, England, U.K) at 1:500. After 3 washes (10 min) in TBS, 0.2% Tween-20, 1M NaCl, the membranes were incubated for 1 hour at room temperature with secondary antibodies diluted in blocking solution: anti-mouse HRP conjugated antibody (Jackson 115-055-068, 1:10,000), ECL Plex anti-mouse Cy3 conjugated (GE Healthcare #PA43009V, 1:2,500), ECL Plex anti-rabbit Cy5 conjugated (GE Healthcare #PA45011V, 1:2,500). After washes in blocking solution, in TBS, 0.2% Tween-20 then TBS, membranes were revealed by ECL (Clarity Biorad chemiluminescence kit # 170–5061) according to the manufacturer’s instructions) or direct fluorescence detection on a LAS4010 (GE Healthcare #28-9558-11) with R670 Cy5 filter, 575DF20 Cy3 filter, according to the manufacturer’s instructions.

### LC MS/MS and phosphorylation analysis

*T*. *brucei brucei* PCF 427 29–13 whole cell extracts was run on a 12% SDS-PAGE and stained with colloidal blue. After several H_2_O washes, a 60–80KDa band was excised and trypsin digested before LC-MS/MS analysis. Using Discoverer 1.3 (PhosphoRS module), one phosphorylation was identified on serine 163 (HAsFHGSTSNALVPR).

## Supporting Information

S1 Fig BILBO1 forms helical polymers when expressed in a heterologous system. Heterologous expression of un-tagged BILBO1 in mammalian U-2 OS cells demonstrates that BILBO1 has self-polymerizing properties. Un-tagged full-length BILBO1 protein was immuno-labelled with anti-BILBO1 monoclonal antibody 24 hours after transfection. In this image long helical polymers are formed within the cell. (A) DAPI (blue) and immunofluorescence (red) merged image of full-length BILBO1 polymers. (B) Enlarged immunofluorescence image of full-length BILBO1 polymers. (C) Phase contrast image of the same polymers observed in B. (D). Phase contrast and fluorescence-merged images B and C. Scale bars represent 10 μm in A and 1 μm in B.
(TIF)

S2 Fig BILBO1 polymers are formed independently of the Golgi, ER, or the cytoskeleton when expressed in U-2 OS cells. U-2 OS cells expressing BILBO1-GFP for six hours were probed or immuno-labelled with cellular markers. F-Actin was probed with Texas red-coupled phalloidin (A-C), intermediate filaments were labelled with anti-vimentin (D-F), microtubules were labelled with anti-tubulin (G-I), the Golgi apparatus was labelled with anti-giantin (J-L), and the endoplasmic reticulum was labelled with anti-calnexin (M-O). Scale bar represents 10 μm. No apparent co-localization of BILBO1-GFP with any of these structures was observed.
(TIF)

S3 Fig mEFH1+2 protein is degraded in U-2 OS cells. (A) U-2 OS cells expressing mEFH1+2 for six hours were treated with 50μM of the proteasome inhibitor MG132 for six hours, then extracted, fixed and processed for immunofluorescence using anti-NTD. (B) The graph shows the percentage of cells in the MG132 experiment that retained anti-NTD signal. (C) U-2 OS whole cells (WC) that were expressing mEFH1+2 were MG132 treated (+) or mock treated (-) and subject to western blotting using anti-BILBO1 5F3B3. Quantification of the western-blot and tubulin normalization indicates and increase in protein level in MG132 treated cells.
(TIF)

S4 Fig Impact on the overexpression of myc tagged recombinant forms of BILBO1 on endogenous BILBO1 levels in *T*. *brucei*, and effect of the proteasome inhibitor on mEFH1+2:myc protein levels. Western blot analysis of overexpression of myc tagged recombinant forms of BILBO1 on endogenous BILBO1 levels in *T*. *brucei*. Endogenous BILBO1 levels was quantified in *T*. *brucei* cytoskeletons derived from cell lines expressing recombinant T1:myc, T2:myc (A), T3:myc, T4:myc (B), BILBO1:myc, mEFH1:myc, mEFH2:myc, and mEFH1+2:myc (C). All samples were tested for six or 24 hours. In (C) the NTD antibody was able to define the difference between wild-type and myc tagged protein due to the higher molecular mass of the myc tagged form. Therefore in the upper panel of (C) wild-type protein is present as the lower band and myc tagged protein is the upper band. (D) *T*. *brucei* mEFH1+2:myc expressing cells were mock treated (-) or treated with 42 μM MG132 (+). Quantification analyses were done using tubulin as loading control (probed with TAT1). Anti-NTD labels endogenous BILBO1, BILBO1:myc, T1:my, T2:myc, mEFH1:myc, mEFH2:myc, and mEFH1+2:myc.
(TIF)

S5 Fig Yeast two-hybrid analysis of FPC5-BILBO1 EF-hand mediated interaction. (A) Yeast two-hybrid analysis indicates that full-length BILBO1 interacts with full-length BILBO1, and a deleted EF-hand form of BILBO1 where the N-terminal domain is retained ΔEFH1+2). We also tested mutant forms of both EF-hands (mEFhand1+2) versus the coiled-coil domain of BILBO1 (T4), or the N-terminal deleted form of BILBO1 (T3). (B) Full-length BILBO1 interacts with the binding domain of FPC5 (FPC5_binding domain_), whilst deletion of both EF-Hands (ΔEFH1+2) or mutation of both EF-Hands (mEHH1+2) prevents this interaction. BILBO1 and FPC5_binding domain_, were tested both as bait (AD) or prey (BD) and demonstrate that EF-hands are required for BILBO1-FPC5_binding domain_. Yeast transformants expressing the combinations of constructs indicated in the figure were spotted onto plates without or with histidine (-His and +His, respectively). Bait and prey interactions were tested by drop test (10^5^cells) and incubated at 30°C for 3 days before analysis.
(TIF)

S6 Fig (A) Determination of the percentage of simple or complex fibres in BILBO1 U-2 OS cells after six or 24 hours post-transfection with or without BAPTA-AM treatment (25ug/ml, for three hours). No significant difference was observed between treated and untreated cells. (B) Immunofluorescence labelling of cytoskeletons from cells expressing mEFH1:myc for six hours and then treated with 5mM EGTA for 10 minutes before fixation and processing. Cytoskeletons were probed using anti-myc (red) and anti-NTD (green) antibodies and show that the polymers were not extracted by EGTA treatment. Scale bars represent 5 μm.
(TIF)
