# Correction: BILBO1 Is a Scaffold Protein of the Flagellar Pocket Collar in the Pathogen *Trypanosoma brucei*

**DOI:** 10.1371/journal.ppat.1004844

**Published:** 2015-04-14

**Authors:** Célia Florimond, Annelise Sahin, Keni Vidilaseris, Gang Dong, Nicolas Landrein, Denis Dacheux, Anna Albisetti, Edward H. Byard, Mélanie Bonhivers, Derrick R. Robinson

The following information is missing from the Funding section: C.F. was funded by a Bourse Region Martinique. This research was funded by an ANR Blanc "Biopocket", and by The Laboratoire d'Excellence (LabEx) ParaFrap ANR-11-LABX-0024 (http://www.agence-nationale-recherche.fr/investissements-d-avenir/appels-a-projets/2011/laboratoires-d-excellence/).
