# Supplementary figures and images for: BILBO1 Is a Scaffold Protein of the Flagellar Pocket Collar in the Pathogen Trypanosoma brucei
Source: PLoS Pathog. 2015 Mar 30;11(3):e1004654. doi: 10.1371/journal.ppat.1004654 (PMC4379179; doi:10.1371/journal.ppat.1004654)

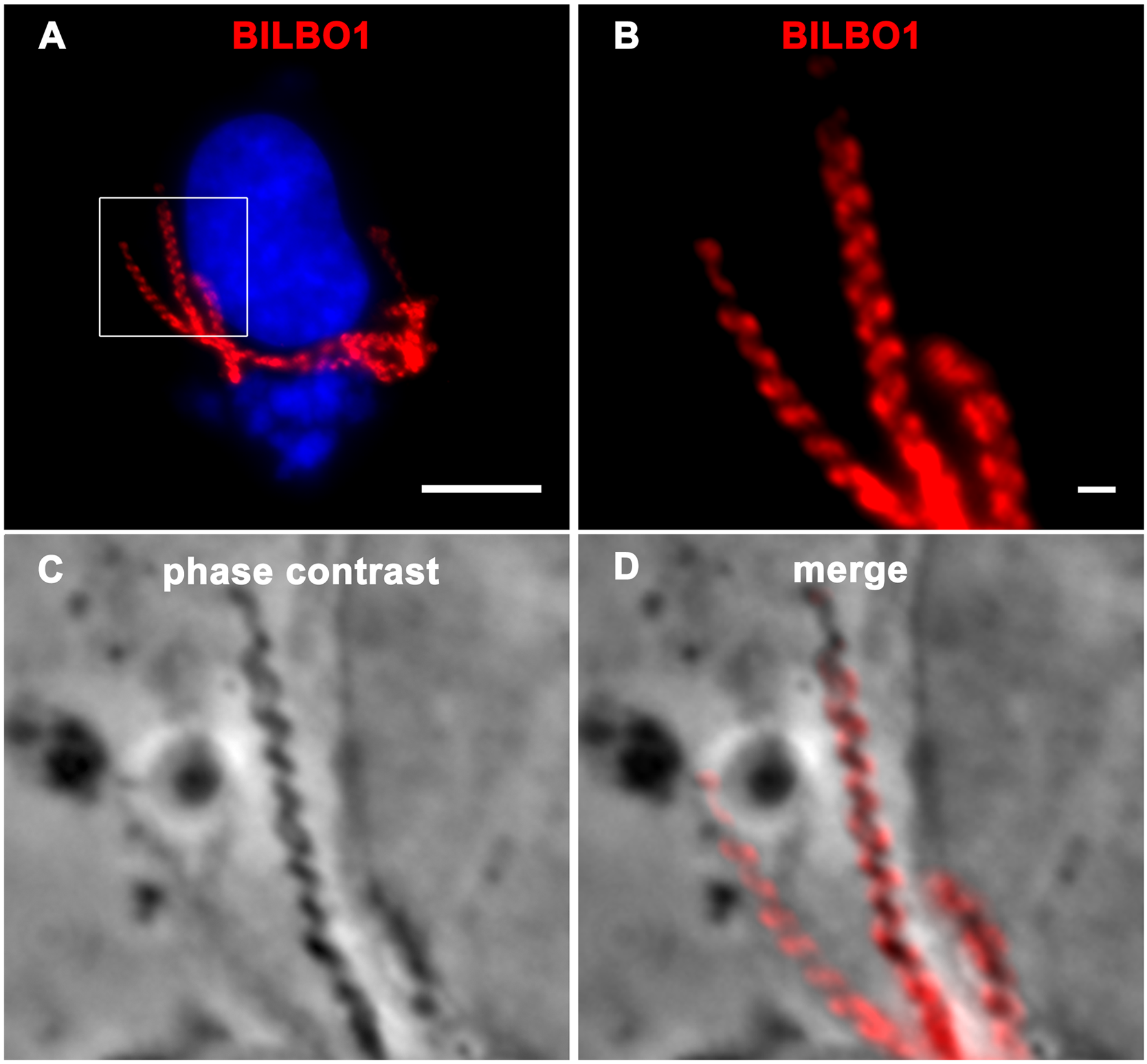

Supplement: S1 Fig — Heterologous expression of un-tagged BILBO1 in mammalian U-2 OS cells demonstrates that BILBO1 has self-polymerizing properties. Un-tagged full-length BILBO1 protein was immuno-labelled with anti-BILBO1 monoclonal antibody 24 hours after transfection. In this image long helical polymers are formed within the cell. (A) DAPI (blue) and immunofluorescence (red) merged image of full-length BILBO1 polymers. (B) Enlarged immunofluorescence image of full-length BILBO1 polymers. (C) Phase contrast image of the same polymers observed in B. (D). Phase contrast and fluorescence-merged images B and C. Scale bars represent 10 μm in A and 1 μm in B. (TIF) [file ppat.1004654.s001.tif]

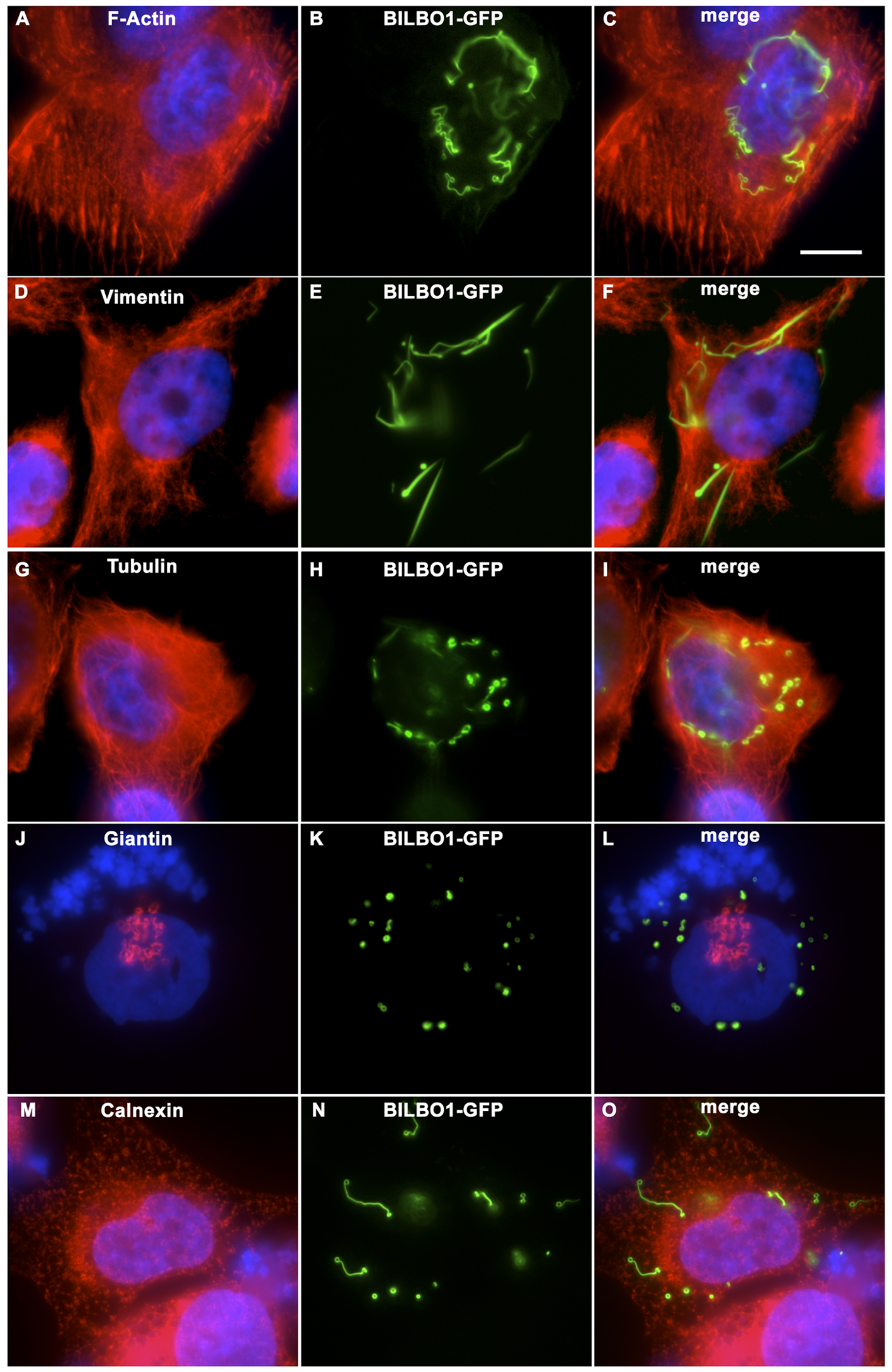

Supplement: S2 Fig — U-2 OS cells expressing BILBO1-GFP for six hours were probed or immuno-labelled with cellular markers. F-Actin was probed with Texas red-coupled phalloidin (A-C), intermediate filaments were labelled with anti-vimentin (D-F), microtubules were labelled with anti-tubulin (G-I), the Golgi apparatus was labelled with anti-giantin (J-L), and the endoplasmic reticulum was labelled with anti-calnexin (M-O). Scale bar represents 10 μm. No apparent co-localization of BILBO1-GFP with any of these structures was observed. (TIF) [file ppat.1004654.s002.tif]

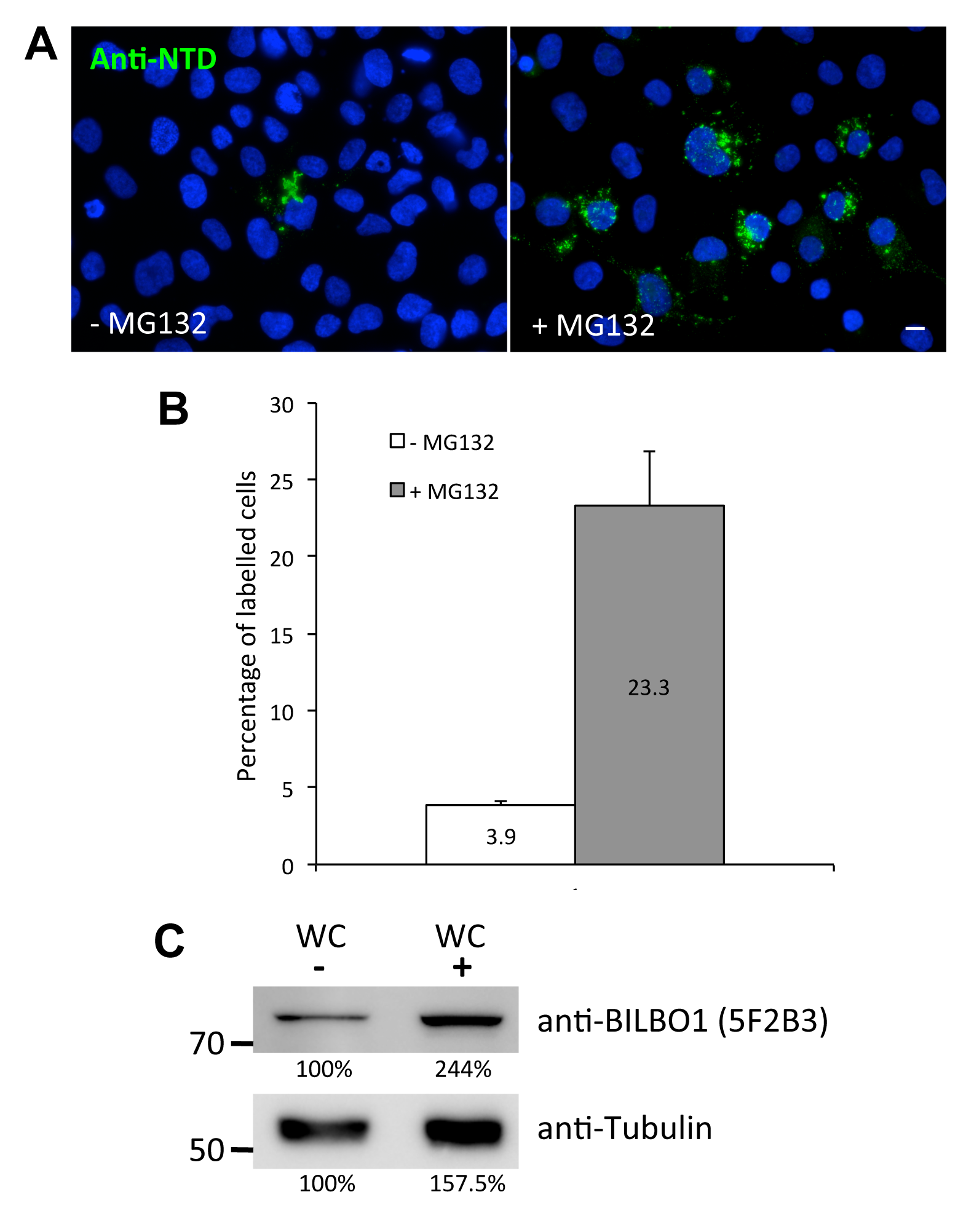

Supplement: S3 Fig — (A) U-2 OS cells expressing mEFH1+2 for six hours were treated with 50μM of the proteasome inhibitor MG132 for six hours, then extracted, fixed and processed for immunofluorescence using anti-NTD. (B) The graph shows the percentage of cells in the MG132 experiment that retained anti-NTD signal. (C) U-2 OS whole cells (WC) that were expressing mEFH1+2 were MG132 treated (+) or mock treated (-) and subject to western blotting using anti-BILBO1 5F3B3. Quantification of the western-blot and tubulin normalization indicates and increase in protein level in MG132 treated cells. (TIF) [file ppat.1004654.s003.tif]

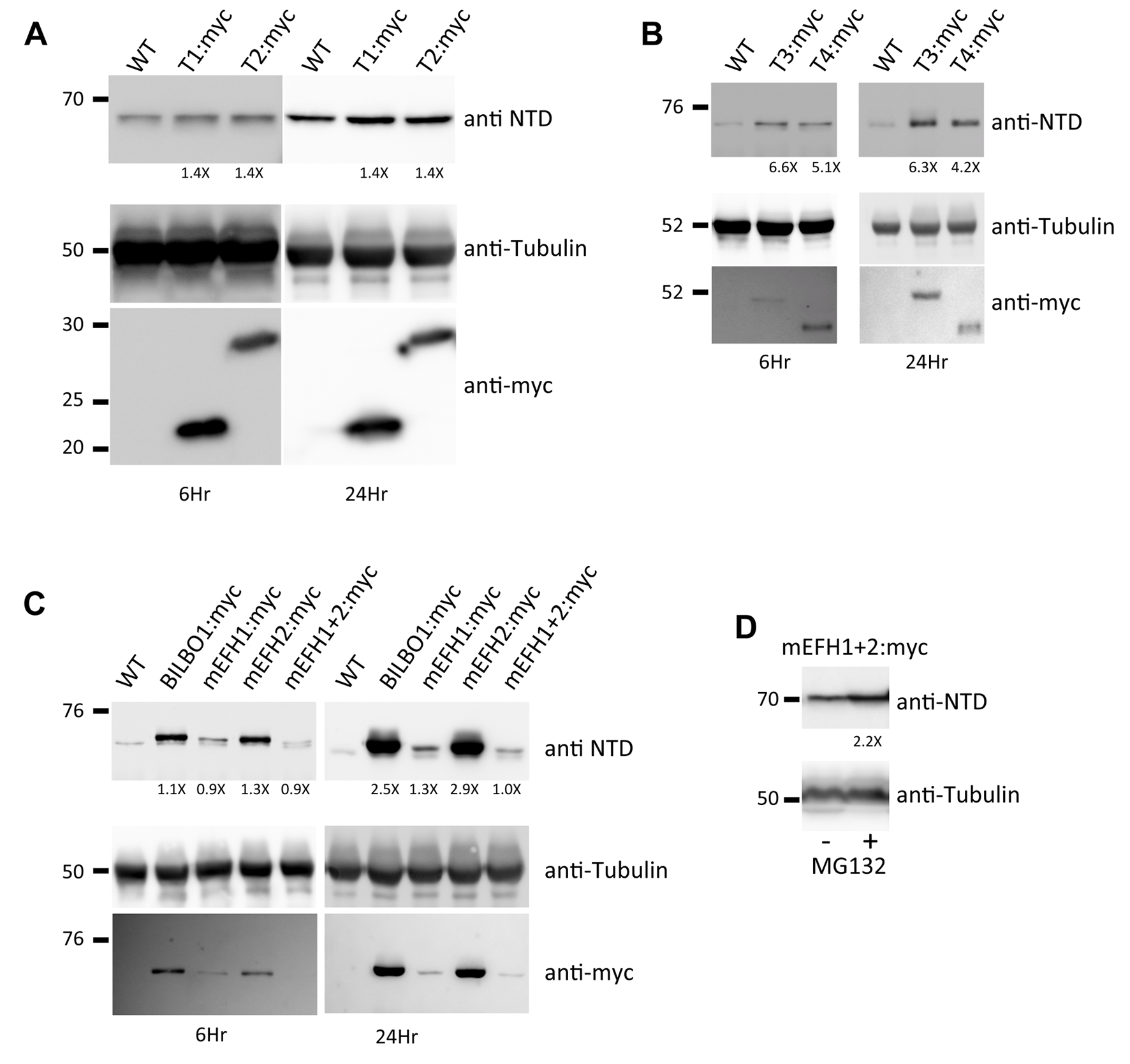

Supplement: S4 Fig — Western blot analysis of overexpression of myc tagged recombinant forms of BILBO1 on endogenous BILBO1 levels in T. brucei. Endogenous BILBO1 levels was quantified in T. brucei cytoskeletons derived from cell lines expressing recombinant T1:myc, T2:myc (A), T3:myc, T4:myc (B), BILBO1:myc, mEFH1:myc, mEFH2:myc, and mEFH1+2:myc (C). All samples were tested for six or 24 hours. In (C) the NTD antibody was able to define the difference between wild-type and myc tagged protein due to the higher molecular mass of the myc tagged form. Therefore in the upper panel of (C) wild-type protein is present as the lower band and myc tagged protein is the upper band. (D) T. brucei mEFH1+2:myc expressing cells were mock treated (-) or treated with 42 μM MG132 (+). Quantification analyses were done using tubulin as loading control (probed with TAT1). Anti-NTD labels endogenous BILBO1, BILBO1:myc, T1:my, T2:myc, mEFH1:myc, mEFH2:myc, and mEFH1+2:myc. (TIF) [file ppat.1004654.s004.tif]

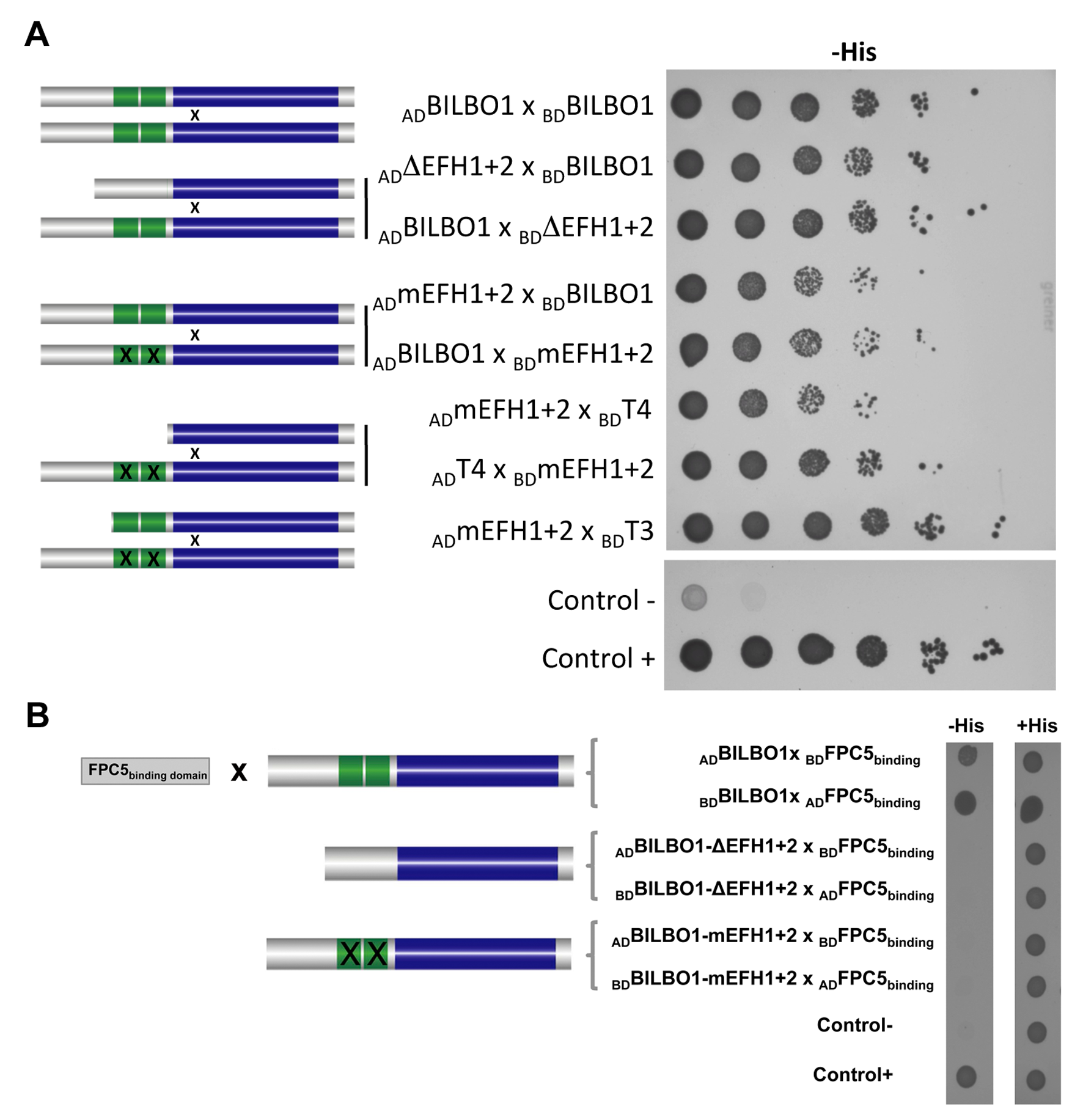

Supplement: S5 Fig — (A) Yeast two-hybrid analysis indicates that full-length BILBO1 interacts with full-length BILBO1, and a deleted EF-hand form of BILBO1 where the N-terminal domain is retained ΔEFH1+2). We also tested mutant forms of both EF-hands (mEFhand1+2) versus the coiled-coil domain of BILBO1 (T4), or the N-terminal deleted form of BILBO1 (T3). (B) Full-length BILBO1 interacts with the binding domain of FPC5 (FPC5binding domain), whilst deletion of both EF-Hands (ΔEFH1+2) or mutation of both EF-Hands (mEHH1+2) prevents this interaction. BILBO1 and FPC5binding domain, were tested both as bait (AD) or prey (BD) and demonstrate that EF-hands are required for BILBO1-FPC5binding domain. Yeast transformants expressing the combinations of constructs indicated in the figure were spotted onto plates without or with histidine (-His and +His, respectively). Bait and prey interactions were tested by drop test (105cells) and incubated at 30°C for 3 days before analysis. (TIF) [file ppat.1004654.s005.tif]

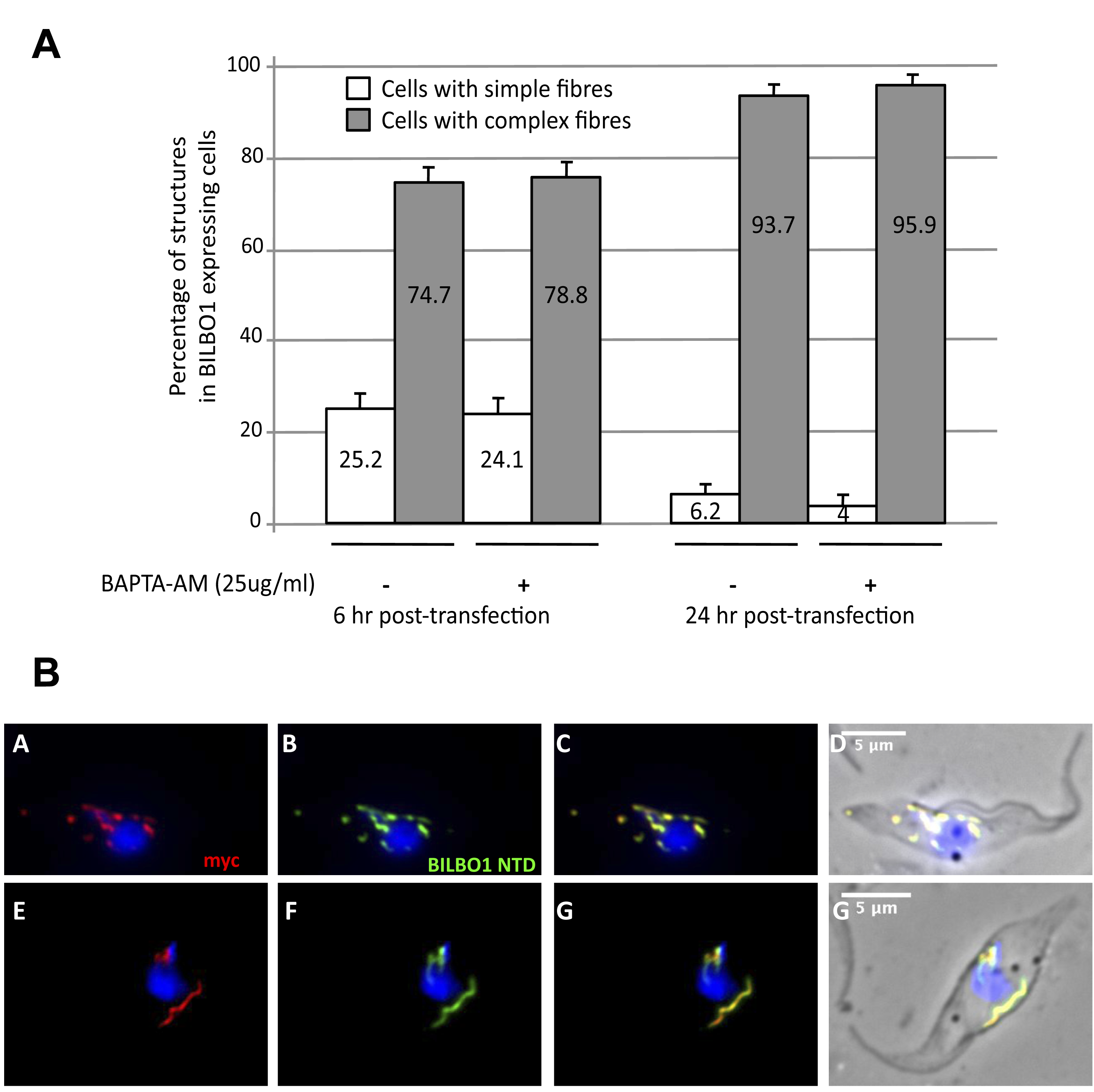

Supplement: S6 Fig — No significant difference was observed between treated and untreated cells. (B) Immunofluorescence labelling of cytoskeletons from cells expressing mEFH1:myc for six hours and then treated with 5mM EGTA for 10 minutes before fixation and processing. Cytoskeletons were probed using anti-myc (red) and anti-NTD (green) antibodies and show that the polymers were not extracted by EGTA treatment. Scale bars represent 5 μm. (TIF) [file ppat.1004654.s006.tif]
